# Within-host infectious disease models accommodating cellular coinfection, with an application to influenza^†^

**DOI:** 10.1093/ve/vez018

**Published:** 2019-07-08

**Authors:** Katia Koelle, Alex P Farrell, Christopher B Brooke, Ruian Ke

**Affiliations:** 1 Department of Biology, Emory University, 1510 Clifton Rd #2006, Atlanta, GA, USA; 2 Department of Mathematics, North Carolina State University, 2311 Stinson Dr, Raleigh, NC, USA; 3 Department of Mathematics, University of Arizona, 617 N Santa Rita Ave, Tucson, AZ, USA; 4 Department of Microbiology, University of Illinois at Urbana-Champaign, 601 S. Goodwin Ave, IL, USA; 5 Carl R. Woese Institute for Genomic Biology, University of Illinois at Urbana-Champaign, 601 S. Goodwin Ave, IL, USA; 6 Comparative Medicine Institute, North Carolina State University, Raleigh, NC, USA

**Keywords:** influenza virus, within-host dynamics, macroparasite model, cellular coinfection, viral complementation

## Abstract

Within-host models are useful tools for understanding the processes regulating viral load dynamics. While existing models have considered a wide range of within-host processes, at their core these models have shown remarkable structural similarity. Specifically, the structure of these models generally consider target cells to be either uninfected or infected, with the possibility of accommodating further resolution (e.g. cells that are in an eclipse phase). Recent findings, however, indicate that cellular coinfection is the norm rather than the exception for many viral infectious diseases, and that cells with high multiplicity of infection are present over at least some duration of an infection. The reality of these cellular coinfection dynamics is not accommodated in current within-host models although it may be critical for understanding within-host dynamics. This is particularly the case if multiplicity of infection impacts infected cell phenotypes such as their death rate and their viral production rates. Here, we present a new class of within-host disease models that allow for cellular coinfection in a scalable manner by retaining the low-dimensionality that is a desirable feature of many current within-host models. The models we propose adopt the general structure of epidemiological ‘macroparasite’ models that allow hosts to be variably infected by parasites such as nematodes and host phenotypes to flexibly depend on parasite burden. Specifically, our within-host models consider target cells as ‘hosts’ and viral particles as ‘macroparasites’, and allow viral output and infected cell lifespans, among other phenotypes, to depend on a cell’s multiplicity of infection. We show with an application to influenza that these models can be statistically fit to viral load and other within-host data, and demonstrate using model selection approaches that they have the ability to outperform traditional within-host viral dynamic models. Important *in vivo* quantities such as the mean multiplicity of cellular infection and time-evolving reassortant frequencies can also be quantified in a straightforward manner once these macroparasite models have been parameterized. The within-host model structure we develop here provides a mathematical way forward to address questions related to the roles of cellular coinfection, collective viral interactions, and viral complementation in within-host viral dynamics and evolution.

## 1. Introduction

In part through the development and analysis of mathematical models, the processes driving the within-host dynamics of viral infections have been increasingly well understood over the last two decades. Statistical fitting of models to within-host data such as viral load measurements and immune response data have yielded estimates of within-host basic reproduction numbers for various viral pathogens, including HIV (Ribeiro et al. 2010), influenza (Baccam et al. 2006; Saenz et al. 2010; Pawelek et al. 2012), measles (Heffernan and Keeling 2008), and dengue (Ben-Shachar and Koelle 2014; Clapham et al. 2014). These fits have further characterized the roles of the innate immune response (Saenz et al. 2010; Pawelek et al. 2012; Ben-Shachar and Koelle 2014), and, particularly in secondary infections, the adaptive immune response (Handel, Longini, and Antia 2010; Ben-Shachar and Koelle 2014) in regulating within-host viral dynamics. The structure of these within-host models has generally mirrored the structure of epidemiological ‘microparasite’ models, with cells being considered either uninfected or infected. In some models (Saenz et al. 2010; Pawelek et al. 2012), uninfected cells have been further categorized as either susceptible or refractory to infection, again, mirroring hosts who are either susceptible or immune to infection in epidemiological models.

While these within-host models capture many of the important features of within-host viral processes, the majority of them implicitly assume that cellular coinfection does not occur (Smith and Perelson 2011) or that cellular coinfection, if it occurs, does not affect the phenotypes of infected cells (Dixit and Perelson 2004; Phan and Wodarz 2015). Yet several experimental findings point toward cellular multiplicity of infection (MOI) having the potential to impact cellular phenotypes such as the rate at which infected cells produce viral output (White et al. 1965; White and Cheyne 1966), the duration of the eclipse phase (Dou et al. 2017), and the probability of a cell initiating an interferon response (Gifford 1963). The implicit assumption that a cell’s MOI does not impact its phenotypes is hard-wired into ‘microparasite’-structured models because these models generally only consider a single class of infected cells, regardless of cellular MOI. With increasing genomic evidence that cellular coinfection frequently occurs in chronic viral infections such as HIV (Onafuwa-Nuga and Telesnitsky 2009) and hepatitis C virus (Ke et al. 2018), as well as in acute viral infections such as influenza (Marshall et al. 2013; Brooke et al. 2014; Fukuyama et al. 2015), a few notable models have been developed that have accommodated the possibility of cellular coinfection (Dixit and Perelson 2004, 2005; Wodarz and Levy 2009, 2011; Phan and Wodarz 2015). However, these models either remain high dimensional (Phan and Wodarz 2015) or have made the assumption that host cell resources are limiting, such that viral output is independent of the extent of cellular coinfection (Dixit and Perelson 2005). While this assumption may be warranted for some viruses, it is likely not met in the case of many other viral pathogens.

Here, we develop a new class of low-dimensional within-host models whose structure flexibly allows for cellular coinfection. We base this new class of models on the structure of epidemiological ‘macroparasite’ models (Roberts, Smith, and Grenfell 1995). Development of these powerful epidemiological models started in the 1970s (Anderson and May 1978, 1992), and they are now being commonly used to study how macroparasites (such as nematodes) spread through host populations (e.g. see Hollingsworth et al. 2015). They have further been used to assess the effect of control strategies on disease burden and host mortality (Truscott, Hollingsworth, and Anderson 2014). We specifically develop this class of within-host ‘macroparasite’ models in the context of acute viral infections, although their structure can also easily accommodate the within-host dynamics of chronic infections. Finally, to demonstrate the usability of these models, we fit specific instances of these models to a classic within-host equine influenza dataset that has previously been analyzed with existing within-host ‘microparasite’ influenza models (Saenz et al. 2010; Pawelek et al. 2012). By performing model selection, we show that the macroparasite models developed here can outperform existing models. These results demonstrate that this new class of models is a viable alternative to traditional within-host ‘microparasite’ models.

## 2. Methods

**Figure 1.**
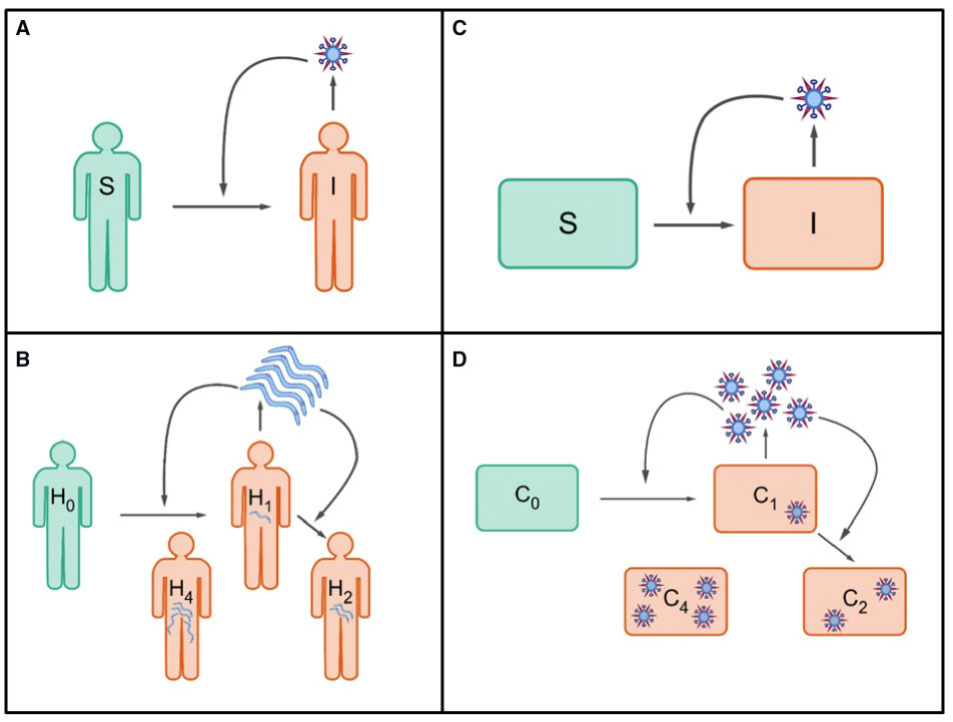
A schematic showing parallels between epidemiological and within-host infectious disease models. Epidemiological models fall into two groups: (A) models for microparasites and (B) models for macroparasites, such as nematodes. Models for microparasites categorize individuals as being infected or uninfected. Models for macroparasites consider the parasite burden of infected individuals, as this burden affects the production rate of macroparasites from infected hosts and the mortality rate of hosts. (C) General structure of current within-host disease models. These models generally categorize cells as being infected or uninfected. (D) Schematic of a within-host ‘macroparasite’ model, proposed here. Models of this type would consider the multiplicity of cellular infection, as multiplicity of infection affects the rate of viral production and the lifespan of infected cells, among other phenotypes.

The structure of the within-host viral dynamic models we propose is based on a close analogy to population-level macroparasite models that are well-established and frequently used in the field of disease ecology and epidemiology (Fig. 1). In Supplementary Text S1, we briefly review the derivation of the canonical structure of these population-level macroparasite models. Co-opting this canonical formulation for within-host viral dynamics allows us to flexibly model cells that have become infected with 0, 1, …, *n* viral particles, while maintaining a low-dimensional set of equations.

### 2.1 Target-cell limited macroparasite models

The simplest version of the within-host macroparasite model is a target-cell limited model. In its most general form, this model is given by:

(1) $$\frac{dH}{dt}=a-\mathrm{bH}-H\sum_{i=0}^{\infty }\alpha_{i}p_{i},$$

(2) $$\frac{dV}{dt}=H\sum_{i=0}^{\infty }\lambda_{i}p_{i}-\eta V-\beta \mathrm{HV},$$

(3) $$\frac{dP}{dt}=\beta \mathrm{HV}-\mathrm{bH}\sum_{i=0}^{\infty }ip_{i}-H\sum_{i=0}^{\infty }{i\alpha }_{i}p_{i}.$$

The variable *H* quantifies the total number of target cells, which includes both uninfected and variably infected cells. In this model, both uninfected and infected cells can be targets of further infection, so this variable differs from the variable representing uninfected target cells in traditional within-host microparasite models. In Equation (1), *a* is the constant rate of target cell production and *b* is the per capita background mortality of target cells. In the absence of infection, the target cell population equilibrates to *H *=* a*/*b*. The third term,$-H\sum_{i=0}^{\infty }\alpha_{i}p_{i}$, is the decrease in the number of target cells due to virus-induced mortality. Here, *α*_*i*_ is the death rate of cells that are infected with a cellular MOI of *i*, and $p_{i}$ is the proportion of target cells that are infected with a cellular MOI of *i*. As such, $p_{i}$ over all cellular MOIs (i∈[0,∞)), is a probability mass function that describes the distribution of cells infected with zero viral particles, one viral particle, two viral particles, etc.

The variable *V* quantifies the amount of free (extracellular) virus, and is analogous to the free virus variable generally modeled in traditional within-host microparasite models. The first term, $H\sum_{i=0}^{\infty }\lambda_{i}p_{i}$, quantifies the overall rate at which free virus is produced from target cells, where $\lambda_{i}$ is the rate at which cells infected with an MOI of *i* produce free virus, and $p_{i}$ again quantifies the proportion of the target cell population that is infected with *i* viral particles. The second term quantifies the rate of viral clearance, and the third term captures loss of free virus from its entry into target cells. This third term is often lost in traditional within-host microparasite models, with an argument that loss of free virus from cell entry is negligible relative to loss of free virus through viral clearance (Smith and Perelson 2011).

The variable *P* quantifies the total amount of internalized virus across all target cells *H* and does not have an analog in traditional within-host microparasite models. This variable is related to, but distinct from, cellular MOI. While cellular MOI quantifies the number of viral particles a single cell has internalized, the variable *P* quantifies the number of internalized viral particles across all existing target cells. The first term in Equation (3) captures the increase in the number of internalized virions from the entry of free virus *V* into target cells *H*. The second term, $\mathrm{bH}\sum_{i=0}^{\infty }ip_{i}$, captures loss of internalized virus through background mortality of target cells. Here, each cell, regardless of cellular MOI, dies at a rate *b*. When a cell with *i* internalized viral particles dies, this results in the loss of *i* internalized virions, such that the overall rate at which internalized particles *P* are lost from the system through background mortality is given by $\mathrm{bH}\sum_{i=0}^{\infty }ip_{i}$. The third term, $H\sum_{i=0}^{\infty }{i\alpha }_{i}p_{i}$, captures the loss of internalized virus through virus-induced mortality. Here, a cell infected with *i* viral particles dies at a rate $\alpha_{i}$. When it dies, its *i* internalized viral particles are lost from the system. As such, across the entire system, the overall rate at which internalized particles *P* are lost through virus-induced mortality is given by $H\sum_{i=0}^{\infty }{i\alpha }_{i}p_{i}$.

At this point, we can further simplify the model by adopting specific assumptions. For instance, in an acute viral infection, the rate of target cell production *a* and the background rate of cell mortality *b* are frequently assumed to be small, such that the terms that include these parameters can be ignored. We can also make certain assumptions about how infected cell phenotypes, such as virus-induced cellular death rates and virus production rates, scale with cellular MOI. For example, we can make analogous assumptions to the ones made in epidemiological macroparasite models, specifically, that the viral production rate is linearly related to cellular MOI ($\lambda_{i}=\lambda i$, where *λ* is now a scalar constant), and that the cell mortality rate scales linearly with cellular MOI ($\alpha_{i}=\alpha i$, where *α* is now also a scalar constant). Applying these specific assumptions, Equations (1–3) become the following for an acute infectious disease:

(4) $$\frac{dH}{dt}=-\alpha P,$$

(5) $$\frac{dV}{dt}=\lambda P-\eta V-\beta \mathrm{HV},$$

(6) $$\frac{dP}{dt}=\beta \mathrm{HV}-\alpha H\sum_{i=0}^{\infty }{i^{2}p}_{i}.$$

To further simplify Equation (6), we can again adopt an analogous assumption to one that is present in epidemiological macroparasite models: that the distribution of cellular MOIs is given by a negative binomial distribution with mean *P*/*H* and dispersion parameter *k* in the range of (0, ∞). This assumption simplifies Equation (6) to:

(7) $$\frac{dP}{dt}=\beta \mathrm{HV}-\alpha P-\alpha \frac{\left(1+k\right)}{k}\frac{P^{2}}{H}.$$

The negative binomial distribution allows for the possibility of cellular MOI overdispersion (low *k*), while still allowing for a Poisson distribution of cellular MOIs when *k* = ∞. Overdispersion of cellular MOIs *in vivo* is highly likely for several reasons. First, some target cells might be more susceptible to infection than others due to variation in the number and types of receptors. Second, given spatial aspects of within-host viral spread, there is likely considerable variation in the rate at which cells are exposed to virus. Third, variation in the time cells remain in their eclipse phase can under certain conditions produce overdispersion of cellular MOIs (Supplementary Text S2).

We can define the within-host basic reproduction number *R*_0_ for the specific target-cell limited model given by Equations (4), (5), and (7) as the number of new, successfully internalized viral particles generated by a single internalized viral particle at the onset of an individual’s infection when the overwhelming majority of target cells are uninfected. To derive *R*_0_ for this model, we can first make a fast viral dynamics assumption, such that $\frac{dV}{dt}\approx 0$ and $V\approx \frac{\lambda P}{\eta +\beta H}$. Plugging this expression into Equation (7), and recognizing that the ratio $\frac{P^{2}}{H}\approx 0$ at the onset of infection, yields: $\frac{dP}{dt}=\frac{\lambda H}{\eta /\beta +H}P-\alpha P$. From this expression, it is clear that $R_{0}=\frac{\lambda \left[\frac{H_{0}}{\frac{\eta }{\beta }+H_{0}}\right]}{\alpha }$, where $H_{0}$ is the number of target cells present at the beginning of the infection. While we assume fast viral dynamics in the derivation of *R*_0_, we continue to model within-host viral dynamics under the target-cell limited version of this model using all three variables (*H*, *V*, and *P*). We do this because it is uncommon to assume fast viral dynamics in within-host models and retaining *V* in the model allows for a more straightforward interface with viral load data.

Since little is known about how viral production rates and cellular mortality rates scale with cellular input, alternative assumptions can also be made that would still allow for a simplification of Equations (1–3). For example, it could be assumed that viral production rates are independent of cellular input, as long as a cell is infected. This assumption would implicitly assume that host cell machinery is the limiting factor governing viral production from a cell. This assumption would lead to Equation (2) becoming:

(8) $$\frac{dV}{dt}=\lambda H\left(1-{\left(\frac{1}{1+P/(\mathrm{Hk})}\right)}^{k}\right)-\eta V-\beta \mathrm{HV},$$

where the term $\left(1-{\left(\frac{1}{1+P/(\mathrm{Hk})}\right)}^{k}\right)$ provides the probability that a target cell has internalized at least one viral particle. It could also be assumed that the cellular mortality rate is independent of cellular input (as long as there is some input). In this case, Equations (1) and (3) would become:

(9) $$\frac{dH}{dt}=-\alpha H\left(1-{\left(\frac{1}{1+P/(\mathrm{Hk})}\right)}^{k}\right),$$

(10) $$\frac{dP}{dt}=\beta \mathrm{HV}-\alpha P.$$

We can further determine under what set of assumptions this within-host macroparasite model would be equivalent to, or fold into, the structure of a within-host microparasite model. A particularly useful step to demonstrate the mapping between the macroparasite model and the microparasite model is to assume that virions are internalized independently of a cell’s MOI. This assumption would be met under mean-field mixing assumptions (i.e. in the absence of spatial structure) and by ignoring the possibility of superinfection exclusion. In the case of this assumption, the value of the dispersion parameter is *k* = ∞. With viral production rates and virus-induced cell death rates that are independent of cellular MOI, Equations (8) and (9) become:

(11) $$\frac{dV}{dt}=\lambda H\left(1-e^{-P/H}\right)-\eta V-\beta \mathrm{HV}$$

(12) $$\frac{dH}{dt}=-\alpha H\left(1-e^{-P/H}\right),$$

where $e^{-P/H}$ is the (Poisson) probability of a cell not being infected. Defining the number of currently infected target cells as $I=H\left(1-e^{-P/H}\right)$ allows one to expand Equation (12) into uninfected (*T*) and infected (*I*) target cell classes: $\frac{dT}{dt}=-\beta \mathrm{TV}$ and $\frac{dI}{dt}=\beta \mathrm{TV}-\alpha I$, respectively. This definition also allows us to simplify Equation (11) to $\frac{dV}{dt}=\lambda I-\eta V$, with the third term in Equation (11) (βHV) assumed to be negligible. The variable *P* can be excluded if it assumed to be in equilibrium with *V*. As such, it is clear that with the assumption of Poisson-distributed cellular MOIs, an MOI-independent viral production rate, and an MOI-independent mortality rate of infected cells, the within-host macroparasite model folds into the traditional within-host microparasite model. This finding is consistent with findings from a previous, high dimensional model for HIV that accommodated multiply infected cells (Dixit and Perelson 2005). An analysis of that model showed that it simplified to the structure of a within-host microparasite model that had all infected cells belonging to a single infected class *I* when viral production rates (and infected cell mortality rates) were independent of the number of internalized virions (Dixit and Perelson 2005).

The empirical relationship between cellular input and the rate of viral production likely depends on virus and host cell type, and needs to be empirically addressed when applying the within-host macroparasite model to a specific viral infection. Similarly, little is known about how cellular mortality rate scales with cellular input and experimental studies need to be performed to address this major knowledge gap.

### 2.2 Within-host ‘macroparasite’ models incorporating the host’s immune response

Within-host models of viral infections frequently incorporate the host’s immune response, since it is this response that is thought to play a critical role in regulating and ultimately clearing viral infections (Smith and Perelson 2011; Ben-Shachar and Koelle 2014). Minimally, the host’s immune response can be incorporated by considering only the innate immune response, which can, again at minimum, be captured by a single additional variable. This variable can encompass the activity of interferons and cytokines, as well as cells of the innate immune response such as natural killer (NK) cells. In many models, the dynamics of specifically interferon-*α* (IFN-*α*) have been included, with interferon production occurring from infected cells and decaying at a constant rate (Saenz et al. 2010; Pawelek et al. 2012; Ben-Shachar and Koelle 2014). If we assume that cells produce interferon at a rate (or probability) that scales linearly with cellular MOI, then the dynamics of interferon-*α* are given by:

(13) $$\frac{dF}{dt}=\mathrm{qP}-\mathrm{dF}.$$

Generally, measurements of IFN-*α* have been reported in units of fold change. As such, the variable *F* has most commonly been considered to be in units of fold change (Pawelek et al. 2012). While in Equation (13) we assumed that IFN-*α* production scales linearly with cellular MOI, alternative assumptions can again be made, for example, that all infected cells produce interferon at an equal rate, independent of cellular MOI.

IFN-*α* can modify viral within-host dynamics in a number of ways. One way is for it to reduce the rate of viral production from infected cells. Another way is for it to decrease the susceptibility of cells to infection (or further infection). Both of these mechanisms of action can be assumed to respond to immediate levels of interferon. In this case, the viral production rate can be reduced from $H\sum_{i=0}^{\infty }\lambda_{i}p_{i}$ (Equation 2) to $\frac{H}{1+\varepsilon F}\sum_{i=0}^{\infty }\lambda_{i}p_{i}$, or similar (as in Canini and Carrat 2011), and the rate of viral entry into target cells can be reduced from βHV (Equations 2 and 3) to $\frac{\beta }{1+\varepsilon F}HV$ or similar (as in Saenz et al. 2010). Alternatively, cellular exposure to interferon could have prolonged effects, with cells becoming refractory to infection (or further infection) for a period of time (as in Saenz et al. 2010; Pawelek et al. 2012) and infected cells reducing their viral output for a period of time following exposure. A third effect of IFN-*α* is to facilitate the recruitment of innate effector cells, which would act to clear infected target cells, leading to an overall effective increase in the rate at which target cells decline. Here, for simplicity, we consider only two direct effects of interferon: the effect of these molecules on reducing cell susceptibility to infection and on reducing the rate of viral production from infected cells, assuming that interferon has prolonged effects on cells. Our innate immune response model is given by Equation (13) and the following system of equations:

(14) $$\frac{dH}{dt}=-\alpha P-\phi \mathrm{FH}$$

(15) $$\frac{dR}{dt}=\phi \mathrm{FH}$$

(16) $$\frac{dV}{dt}=\lambda P-\eta V-\beta \mathrm{HV}$$

(17) $$\frac{dP}{dt}=\beta \mathrm{HV}-\alpha P-\alpha \frac{\left(1+k\right)}{k}\frac{P^{2}}{H}-\phi \mathrm{FP}.$$

Here, *H* is the total number of currently *susceptible* target cells (including infected and uninfected target cells), *R* is the total number of target cells that are currently *refractory* to further infection (including uninfected cells and already infected cells), *V* is again the amount of free virus, and *P* is the total number of viral particles across *susceptible* target cells. The parameter ϕ quantifies the per capita rate at which interferon makes cells refractory to infection. This model formulation assumes that all susceptible cells, whether uninfected or infected, become refractory to infection (or further infection) at similar rates, that refractory cells stay permanently refractory, and that no virus is produced from refractory cells. This latter assumption effectively reduces the overall rate at which the total infected cell population produces virus as a result of interferon exposure. Model Equations (13–17) assume analogous effects of interferon as the within-host microparasite model presented in Saenz et al. (2010), while adopting assumptions of linear scaling between cellular MOI and infected cell mortality rate, between cellular MOI and viral production rate, and between cellular MOI and the probability of cellular interferon production. The model does not incorporate the possibility for refractory cells to become resusceptible to viral infection within the time period of an acute infection. Further, it does not incorporate the role that other cells of the innate immune response (such as NK cells) may play in clearing infected cells. Finally, it does not incorporate the role that the adaptive immune response may play in terminating the infection. All three of these assumptions have been previously incorporated into a within-host model for influenza infection (Pawelek et al. 2012). We do not incorporate these processes in Equations (13–17) because our goal here is simply to demonstrate through several examples how the within-host macroparasite model can be developed under a set of virological assumptions. We note, however, that in some cases, incorporation of a process into the macroparasite model formulation may be more difficult than into a microparasite model formulation. For example, allowing refractory cells to become resusceptible to infection (as in Pawelek et al. 2012) and to again produce viral output, would be difficult without adding additional variables, since the (currently untracked) distribution of the number of internalized particles within refractory cells is expected to be different from the distribution of the number of internalized particles within susceptible cells.

## 3. Results

Here, we fit the within-host macroparasite models presented above to empirical within-host data, highlighting key quantities that can be calculated from these models that within-host microparasite models have difficulty providing. The within-host data we fit to are from ponies that have been experimentally infected with influenza A virus subtype H3N8. The data have been analyzed in a number of previously published studies (Saenz et al. 2010; Pawelek et al. 2012), and include viral load measurements and IFN-*α* fold change measurements. As a point of comparison, we also consider existing, comparable microparasite model formulations. We start with fitting target cell limited models and then move to considering models that incorporate the immune response.

### 3.1 Target cell limited models

The classic target cell limited microparasite model is given by the following three equations: $\frac{dT}{dt}=-\beta \mathrm{TV}$, $\frac{dI}{dt}=\beta \mathrm{TV}-\alpha I$, and $\frac{dV}{dt}=\lambda I-\eta V$, where *T* is the number of susceptible (and uninfected) target cells, *I* is the number of infected target cells, and *V* is free virus. To estimate model parameters, we use a maximum likelihood approach that accommodates below the limit of detection measurements (Supplementary Text S3). Instead of fitting all parameters and initial conditions individually for each pony, we fix some and estimate others as either group-level or individual-level parameters. Specifically, following Saenz et al. (2010) and Pawelek et al. (2012), across all ponies, we set the initial number of target cells *T*(0) to 3.5 × 10^11^ cells, and the initial number of infected cells *I*(0) to zero. We further constrain the number of model parameters to be estimated by assuming that the viral production rate *λ* is the same across ponies and that the infected cell death rate *α* is the same across ponies. We allow the viral clearance rate *η*, the viral infectivity rate *β*, and the initial viral load *V*(0) all to differ between ponies. Supplementary Table S1 shows maximum likelihood parameter estimates of this target cell limited microparasite model, by pony. We calculated the log-likelihood of the model fit across the ponies to be −55.57. With the number of estimated parameters and initial conditions being twenty-one in all, the akaike information criterion (AIC) score for the model fit was 151.15.

**Figure 2.**
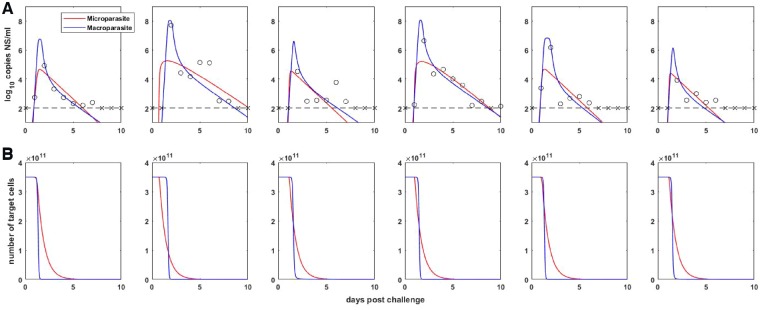
Target-cell limited within-host model dynamics. (A) Within-host viral dynamics, parameterized by fitting target-cell limited models to influenza A/H3N8 viral load measurements from experimentally infected ponies (black circles and x’s). The dashed black line shows the limit of detection, and x markers show below the limit of detection measurements. Colored lines show maximum likelihood fits of the classic within-host target-cell limited microparasite model and of the target-cell limited macroparasite model. (B) The number of target cells over time for the within-host target-cell limited microparasite model (given by *T *+* I*) and for the within-host macroparasite model (given by *H*).

We next fit our target cell limited macroparasite model given by Equations (4), (5), and (7) to these same influenza A/H3N8 viral load measurements. Prior to fitting this model, we confirmed that all of its parameters were structurally identifiable (Supplementary Text S4). Again, due to the limited number of data points for each pony, we did not attempt to estimate all model parameters for each pony independently. Instead, we again set the initial number of target cells (in our case, given by the variable *H*) to 3.5 × 10^11^ cells, and further set the initial number of internalized virions *P* to zero. This latter assumption corresponds to the assumption made in previous models of zero initially infected cells. We further constrained the per-particle production rate *λ*, the per-particle cellular mortality rate *α*, and the dispersion parameter *k* to be the same across infected ponies. We let the viral clearance rate *η*, the viral infectivity rate *β*, and the initial amount of free virus *V*(*0*), differ across ponies, since in part these parameters reflect host-specific characteristics or phenotypes related to a host’s immune history. Using the same maximum likelihood approach, we thus fit twenty-one parameters in total, including initial conditions. Under these constraints, all parameters of this basic model were practically identifiable. Table 1 lists the estimated model parameters, by pony. The within-host basic reproduction number was estimated to be in the range of 16.9–18.8 across the ponies. We calculated the log-likelihood of the model fit across the ponies to be −50.04. With the number of estimated parameters and initial conditions being twenty-one in all, the AIC score for the model fit was 142.07. As such, the target cell limited macroparasite model fit the viral load measurements significantly better than the target cell limited microparasite model. Fig. 2A shows the viral load measurements from the ponies, along with the fit of the classic target cell limited microparasite model and the target cell limited macroparasite model. Fig. 2B shows host target cell dynamics for each of these two models. The fits of the two models show why the macroparasite model was statistically preferred over the microparasite model. The microparasite model does not reproduce the observed biphasic (fast, then slow) viral decline in this dataset and has trouble hitting peak viral loads. Indeed, this target cell limited model has been criticized for its failure to reproduce these two features (Saenz et al. 2010; Pawelek et al. 2012), among other criticisms such as its fundamental assumption that target cells are limiting (Saenz et al. 2010). In contrast, the target cell limited macroparasite model is able to reproduce these two key features of the observed within-host influenza dynamics when fit to the observed data. How robust these two features are across datasets is a separate question that falls outside the scope of the analysis presented here. It is worthwhile to note, however, that there is little indication that humans infected with influenza A virus exhibit high peak viral loads for a short duration of time, or that their viral decline is biphasic, based on data from human H1N1 and H3N2 challenge studies (Baccam et al. 2006; Carrat et al. 2008). And while a recent study in mice also indicated that viral decline is biphasic, it appeared to be characterized by slow, then fast viral decline (Smith et al. 2018), rather than the other way around. Here, we are not suggesting that the patterns observed in this dataset apply to other hosts experiencing infection with influenza A virus, but simply demonstrate that the target cell limited macroparasite model can fit specifically the equine influenza viral load measurements better than the target cell limited microparasite model.

**Table 1.** Parameter estimates for the target cell limited macroparasite model, for each of the six ponies. The model is given by Equations (4), (5), and  (7). “The ponies' within-host basic reproduction numbers are also listed.”

			Pony					
			1	2	3	4	5	6
Parameter or initial condition	Units	Estimated or set	Value					
*H*(0)	Cells	Set	3.50E+11					
log_10_(*V*(0))	log_10_ RNA copies ml^−1^ NS	Estimated (individually)	−2.06	−6.04	−2.75	−3.78	−1.97	−3.02
*P*(0)	Internalized particles	Set	0					
*λ*	RNA copies ml^−1^ NS day^−1^	Estimated (by group)	26.75					
*α*	Day^−1^	Estimated (by group)	1.42					
*k*		Estimated (by group)	0.89					
*η*	Day^−1^	Estimated (individually)	2.91E+03	6.92E+01	1.76E+06	6.44E+02	2.29E+01	3.13E+06
*β*	(RNA copy)^−1^ ml NS day^−1^	Estimated (individually)	7.54E-05	3.73E-06	4.48E-05	3.40E-06	6.57E-05	1.74E-04
*R*_0_			18.84	18.84	16.94	18.83	18.84	17.91

**Figure 3.**
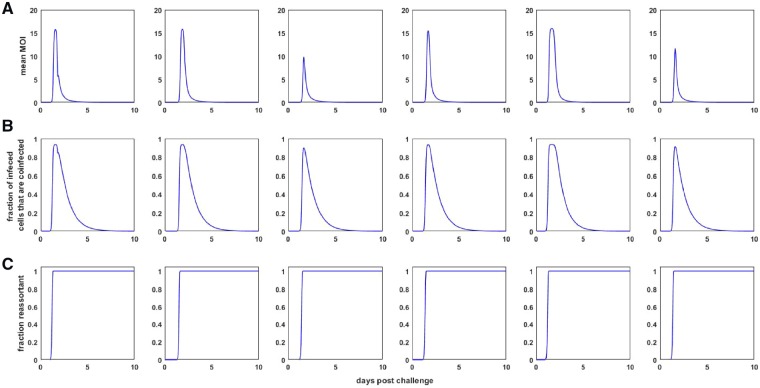
Dynamics of quantities derived from the target-cell limited within-host macroparasite model. (A) Mean multiplicity of infection (MOI) over time for each of the ponies shown in Fig. 2A. Mean MOI is calculated as the total number of intracellular particles divided by the total number of target cells, *P*(*t*)/*H*(*t*), where *t* is time since infection. (B) The proportion of infected cells that are infected by more than one viral particle, calculated from the within-host macroparasite model. (C) The fraction of the viral population that is reassortant, shown over the course of infection.

To understand why the macroparasite model can reproduce these two key features of the observed viral dynamics, we note that the model formulation allows us to easily calculate the time-varying mean MOI (Fig. 3A). The mean MOI in this model is simply given by the total number of internalized particles divided by the total number of target cells: *P*/*H*. Fig. 3A indicates that, as viral load increases, the mean MOI increases dramatically, and therewith the amount of free virus being produced. This allows the high viral peaks to be reproduced. Infected cells with high MOI then experience high mortality rates, leading to a very rapid decline in viral load and a rapid depletion of target cells *H* (Fig. 2B). Mean cellular MOI drops as a result of this rapid depletion of cells with high MOI. It then remains low because of low levels of free virus *V* and thus little opportunity to internalize more virus. The second phase of the viral decline comes about from the low mortality rate of cells with low MOI.

From the formulation of the model, one can also easily calculate the time-varying proportion of infected cells that are infected by more than one viral particle (Fig. 3B). This information may be useful for characterizing the landscape available to defective interfering particles, which ‘cheat’ off of wild-type virus for their own replication (Chao and Elena 2017). One can also project the frequency of reassortants present in the within-host viral population by extending the model given by Equations (4), (5), and (7). Specifically, we can add three additional equations to the target cell limited macroparasite model: $\frac{dH_{0}}{dt}=-\beta H_{0}V$, $\frac{dH_{1,n}}{dt}=\beta H_{0}V_{n}-\alpha H_{1,n}-\beta H_{1,n}V$, $\frac{dV_{n}}{dt}=\lambda H_{1,n}-\eta V_{n}-\beta HV_{n}$. Here, *H*_0_ is the number of uninfected target cells, *H*_1,__*n*_ is the number of target cells that are infected with a single non-reassortant viral particle, and *V_n_* is the non-reassortant free virus population. All other parameters and variables are as in Equations (4), (5), and (7). The initial conditions for these variables are *H*_0_(0) = *H*(0), *H*_1,__*n*_(0) = 0, and *V_n_*(0) = *V*(0). The fraction of the viral population that is reassortant at time *t* is given by $1-\frac{V_{n}(t)}{V(t)}$. Fig. 3C shows the dynamics of this fraction over the course of infection. For the inferred parameter values of the target cell limited macroparasite model, the fraction of the viral population that is reassortant increases rapidly as mean MOI increases, saturating at one. Since barcoding of virus is possible, future studies could thus use frequencies of viral reassortants, quantified from nasal wash samples, as additional data for estimating within-host macroparasite model parameters. Specifics about how this would be done properly would need to be addressed, however, since this model deems all viruses coming out of a coinfected cell as reassortants. If in an infection, a cell is coinfected with two viral particles that have been produced by a single barcoded viral parent, viral output from this cell may appear as non-reassort virus, despite reassortment most likely having taken place.

### 3.2 Within-host models incorporating the immune response

We now turn to fitting the within-host macroparasite model that incorporates the innate immune response, given by Equations (13–17). We compare the fit of this model to those of two previously proposed within-host microparasite models: the model proposed by Saenz et al. (2010) and the one proposed by Pawelek et al. (2012). Again, for our model, we forced a subset of the model parameters to be the same across the ponies, while letting other parameters be pony-specific.

**Figure 4.**
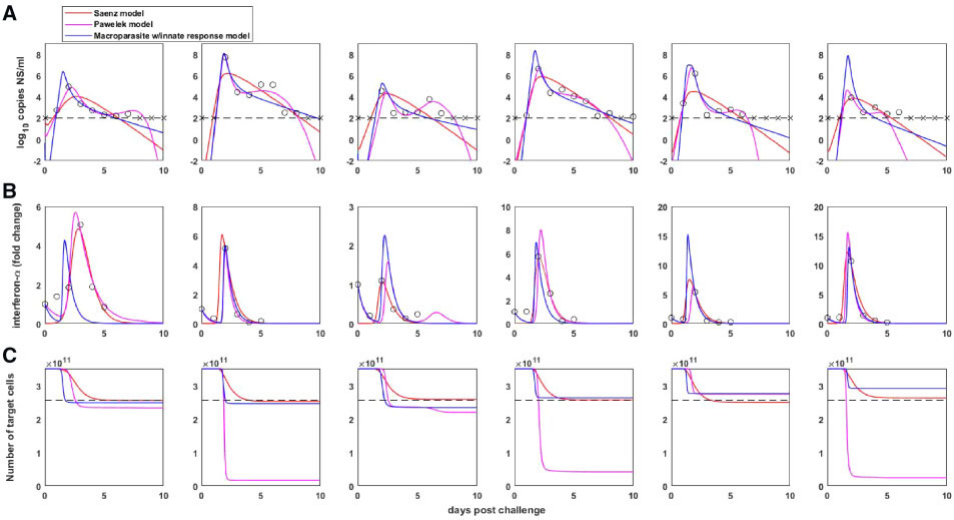
Within-host dynamics from the three considered innate immune response models. (A) Model-simulated within-host viral dynamics, with data and limit of detection shown as in Fig. 2A. (B) Model-simulated interferon-*α* dynamics, along with IFN-*α* fold change measurements. (C) Model-simulated target cell dynamics. Dashed black line shows an estimate for the final number of target cells, given by a 27 per cent reduction in the number of target cells (Saenz et al. 2010).

The Saenz et al. model (Saenz et al. 2010) is an eight-dimensional set of ordinary differential equations with variables *T* (susceptible target cells), *E*_1_ (eclipse phase cells that have not been exposed to interferon), *W* (pre-refractory cells), *E*_2_ (eclipse phase cells that have been exposed to interferon but are not yet refractory), *R* (refractory cells), *I* (infected cells), *V* (free virus), and *F* (interferon). The key component of this model is the introduction of a class of cells that are refractory to viral infection following exposure to interferon. Their model, as structured and parameterized, assumes that IFN-*α* does not affect viral production from already infected cells. The full set of model equations and list of parameters are provided in Saenz et al. (2010). Note that in their analysis, Saenz et al. set initial conditions for interferon-*α F*(0) to zero. Since interferon-*α* measurements are in units of fold change, we (and Pawelek et al.) instead set *F*(0) at one. To estimate their parameters, Saenz et al. used a weighted non-linear least squares procedure that incorporated viral load measurements, IFN-*α* fold change measurements, and an estimate that only 27 per cent of host cells were depleted by the end of an infection. We extended the likelihood function used for fitting the target cell limited models to incorporate both IFN-*α* measurements and the 27 per cent target cell depletion estimate (Supplementary Text S3). Using this likelihood function, we calculated the log-likelihood of the Saenz et al. model to be −255.69 (Table 2). We further calculated the log-likelihood of the Saenz et al. model to be −98.20 when considering only viral load measurements and IFN measurements (Table 2). With the number of estimated parameters and initial conditions being thirty-six in all, the AIC score for the model fit was 583.37 (with target cells in the log-likelihood function) and 268.39 (without target cells in the log-likelihood function). Fig. 4 shows the viral load dynamics, interferon dynamics, and dynamics of the total number of target cells (*T* + *E*_1_ + *W* + *E*_2_ + *R* + *I*) for this model fit.

**Table 2.** Model comparison using AIC. For each of the three models we compare, the table lists the complexity of the model, the models' log-likelihood values (as calculated using the likelihood expressions provided in the Supplementary Material), and AIC values. The boxed cells indicate the preferred model.

Model	Model complexity	Components of likelihood calculation:	Viral load, IFN dynamics	Viral load, IFN dynamics, final target cells
Saenz et al. (2010)	36 estimated parameters	Log-likelihood:	−98.20	−255.69
		AIC:	268.39	583.37
Pawelek et al. (2012)	60 estimated parameters	Log-likelihood:	−92.61	−258.00
		AIC:	305.21	635.99
Macroparasite model with innate immune response	29 estimated parameters	Log-likelihood:	−103.07	−260.69
		AIC:	264.14	579.38

The Pawelek et al. model (Pawelek et al. 2012) is a five-dimensional set of ordinary differential equations with variables *T* (susceptible target cells), *I* (infected cells), *R* (refractory cells), *V* (free virus), and *F* (interferon). The key component of this model is that refractory cells become resusceptible to infection after some amount of time. Further, the model includes an adaptive immune response, which ultimately allows the viral infection to be cleared. The model, as structured, assumes that IFN-*α* does not affect viral production from already infected cells. The full set of model equations and parameter estimates are provided in Pawelek et al. (2012). We noted that the time of the adaptive immune response onset (*μ*) and the estimated initial viral loads were not explicitly listed in the published paper, and therefore requested these from the authors. The values for *μ* are 7, 4, 6, 3, 5, and 4 days post infection, respectively. The values for log_10_(*V*(0)) are 0.63, −8.77, −5.70, −2.04, −2.16, and −7.68, respectively. The authors estimated their model’s parameters by minimizing the root mean square between the data points and model predictions. The data points they considered were viral load measurements and IFN-*α* fold change measurements. They did not use the 27 per cent target cell depletion estimate that Saenz et al. used to fit their model. Using our likelihood function, we calculated the log-likelihood of the Pawelek et al. model to be −258.00 (with target cells in the likelihood function) and −92.61 (without target cells in the likelihood function). With the number of estimated parameters and initial conditions being sixty in all, the AIC score for the model fit was 635.99 (with the target cell component) and 305.21 (without the target cell component) (Table 2). Fig. 4 shows the viral load dynamics, interferon dynamics, and dynamics of the total number of target cells (*T* + *I* + *R*) for this model. Compared to the Saenz et al. model, the Pawelek et al. model better reproduces the viral plateau, as well as the smaller, secondary viral peak that is observed in the majority of the ponies. The ability of the Pawelek model to capture these features is the reason for why the log-likelihood value of the parameterized Pawelek et al. model is higher than that of the Saenz et al. model. The likelihood of the Pawelek et al. model becomes comparable to that of the Saenz et al. model, however, when target cell depletion is incorporated into the likelihood calculation. This is because the second, fourth, and sixth ponies have the majority of their target cells depleted under the parameterized Pawelek et al. model.

We now turn to the macroparasite model incorporating the innate immune response, given by Equations (13–17). This model is a five-dimensional set of ordinary differential equations with variables *H* (target cells), *R* (refractory cells), *P* (internalized viral particles), *V* (free virus), and *F* (interferon). The key component of this model is that any cell exposed to interferon (whether infected or uninfected) will become refractory to further infection. Further, the model assumes that refractory cells are permanently refractory, and while refractory, cells do not produce virus. To fit this model, we constrained the parameters *α*, *ϕ*, *λ*, *d*, and *k* to be the same across the ponies because these parameters quantify infected cell phenotypes that we expect to be common across individuals. We let parameters *η*, *β*, *q*, and *V*(*0*) differ across ponies, due to host-specific factors. We set the other initial conditions to be: *T*(0) = 3.5 × 10^11^ cells as before, *R*(0) = 0, *P*(0) = 0, and *F*(0) = 1. Again, interferon levels are in units of fold change. Table 3 provides parameter estimates for this model, with parameters estimated using the likelihood function that incorporated only viral load measurements and IFN measurements. Notably, the likelihood function that additionally incorporated the 27 per cent target cell depletion estimate gave rise to almost identical parameter values (results not shown). We calculated the log-likelihood of this model fit across the ponies to be −260.69 (with the target cell component) and −103.07 (without the target cell component). With the number of estimated parameters and initial conditions being twenty-nine in all, the AIC scores for the model fit were 579.38 (with the target cell component) and 264.14 (without the target cell component). Both of these AIC scores are significantly lower than those of Pawelek et al. and slightly lower than those of Saenz et al. (Table 2). Fig. 4 shows the viral load dynamics, interferon dynamics, and dynamics of the total number of target cells (*H* + *R*) for this model fit. In Supplementary Fig. S6, we show this model’s dynamics alone (without the Saenz et al. and Pawelek et al. model dynamics). The macroparasite model can quantitatively reproduce features of the viral load and interferon-*α* measurements, without depletion of target cells to unreasonable levels. Here, the very rapid initial viral decline results from several processes: the cellular input-dependent mortality rate of infected cells, the rapid removal of susceptible target cells *H* through exposure to interferon-*α*, and the lack of viral production from refractory cells. The second, slower phase of viral decline results from the slow removal of the remaining infected cells that have low MOI. The within-host basic reproduction number, ignoring the effect of positive IFN levels at the onset of infection, ranged between 20.2 and 31.0 across the ponies. A slightly modified *R*_0_ calculation that takes into consideration that initial interferon fold change levels are set at one leads to a range of *R*_0_ estimates between 9.9 and 15.2. Because the macroparasite model we consider does not incorporate refractory cells becoming resusceptible to further infection, it does not recapitulate the secondary, lower viral peak that is seen in several of the ponies. Incorporating this process would significantly complicate this model, for reasons elaborated upon above. Whether the second viral peak is due to a replenishment of susceptible target cells or due to some other, currently unmodeled, process is an open question.

**Table 3.** Parameter estimates for the within-host ‘macroparasite’ model incorporating the innate immune response, for each of the six ponies. The model is given by Equations (13)–(17).

			Pony					
			1	2	3	4	5	6
Parameter of initial condition	Units	Estimated or set	Value					
*H*(0)	Cells	Set	3.5E+11					
log_10_(*V*(0))	log_10_ RNA copies ml^−1^ NS	Estimated (individually)	−0.35	−6.01	−1.34	−4.19	−3.89	−6.78
*F*(0*)*	Fold change	Set	1					
*R*(0*)*	Cells	Set	0					
*P*(0)	Internalized particles	Set	0					
*λ*	RNA copies ml^−1^ NS day^−1^	Estimated (by group)	30.36					
*α*	Day^−1^	Estimated (by group)	0.98					
*k*		Estimated (by group)	0.30					
*ϕ*	(IFN fold change)^−1^ day^−1^	Estimated (by group)	1.01					
*d*	Day^−1^	Estimated (by group)	1.86					
*η*	Day^−1^	Estimated (individually)	2.46E+06	4.42E+04	3.16E+07	2.21E+04	4.38E+01	3.95E+04
*β*	(RNA copy)^−1^ ml NS day^−1^	Estimated (individually)	2.22E-05	7.92E-07	1.69E-04	3.36E-07	2.86E-05	1.15E-06
*q*	IFN fold change day^−1^ cell^−1^	Estimated (individually)	6.26E-11	7.24E-11	3.08E-11	1.11E-10	2.62E-10	2.91E-10
*R*_0_ (ignoring effect of IFN)			23.54	26.72	20.18	26.08	30.98	28.21
*R*_0_ (considering effect of IFN)			11.59	13.15	9.93	12.84	15.25	13.88

## 4. Discussion

Here, we have developed a new class of within-host models for understanding the *in vivo* dynamics of viral infections. This new class of models differs from existing within-host models in that it allows for target cells to be variably infected and for the degree of cellular input to impact the phenotypes of infected cells, such as their death rate and the rate at which they produce virus. Despite allowing for cellular coinfection dynamics, these models remain low-dimensional. This addresses existing concerns in the literature about the scalability of within-host models that allow for cellular coinfection (Phan and Wodarz 2015). While we have developed and applied these within-host models to acute viral infections, the structure of these models is immediately applicable to chronic viral infections. When applied to chronic infections, the terms we lost for the replenishment and natural death of target cells (see Section 2) would simply need to be reintroduced.

The structure of the within-host models we derived here co-opt the structure of epidemiological macroparasite models. Based on empirical data, those models generally assume that host death rates scale linearly with macroparasite burden and that the rate of egg release from infected hosts similarly scales linearly with macroparasite burden. The within-host models we fit similarly adopt these scaling relationship assumptions, although other scaling assumptions can be adopted while retaining the desirable low-dimensionality of the model equations. Clearly, the structure of the within-host model should reflect empirically supported relationships between cellular input and cellular phenotypes. To date, very few studies have attempted to empirically quantify these relationships, making the appropriate choice of model structure difficult to decide upon. For influenza, the studies that do exist have shown that the timing and amounts of viral yield depend critically on cellular input, and that cumulative viral yield generally increases with cellular input (White et al. 1965; White and Cheyne 1966). Intriguingly, these results stand in contrast to the current structure of the model that we have presented. This is because, if we assume that both the viral production rate and the cell death rate scale linearly with cellular input, the total cellular output of an infected cell should be independent of its MOI *i*, with total cellular output being given by: λi/αi=λ/α. The empirically determined relationship between higher cellular output with higher cellular input therefore seems to indicate that cellular death rates must scale less than linearly with cellular input and/or that viral production rates must scale faster than linearly with cellular input. The latter relationship, if empirically supported, would provide tantalizing evidence for viral cooperation within cells playing a role in within-host viral dynamics, an idea that has recently gained traction (Brooke 2017; Díaz-Muñoz, Sanjuán, and West 2017; Sanjuán 2017).

A key feature of epidemiological macroparasite models is the possibility of nematode overdispersion across hosts. Parasite overdispersion has considerable empirical support, with the overwhelming majority of macroparasite distributions studied having a variance to mean ratio exceeding one and an estimated dispersion parameter *k* of less than one (Shaw and Dobson 1995). A more recent analysis further indicates that observed levels of parasite overdispersion can be attributed almost entirely to host heterogeneity in parasite exposure or host heterogeneity in susceptibility to infection (Poulin 2013). Here, in the context of within-host viral dynamics, we also found statistical support for very high levels of overdispersion, with a dispersion factor *k* estimate of 0.89 in the target-cell limited model (Table 1) and an estimate of *k* = 0.30 in the innate immune response model (Table 3). This overdispersion could similarly reflect variation in target cell susceptibility to infection. It could also reflect heterogeneity in viral exposure, likely due to the intrinsically spatial aspect of influenza virus spread within infected hosts (Gallagher et al. 2018). Finally, as described in Supplementary Text S2, overdispersion could also result from the distribution of time that cells remain in the eclipse phase prior to becoming productively infected. Regardless of the causes of viral overdispersion across target cells, the consequences of viral overdispersion are that the majority of infected cells are multiply infected, at least over some duration of the infection (Fig. 3B). This gives rise to the expectation of considerable levels of viral reassortment within infected hosts (Fig. 3C), consistent with findings from a guinea pig study that found robust reassortment *in vivo* between phenotypically neutral strains that differed from one another only by silent mutations (Marshall et al. 2013). In contrast, however, analysis of viral sequence data from a human challenge study indicated very limited effective reassortment, perhaps because of multiple initiating foci of infection (Sobel Leonard et al. 2017).

In addition to its effects on viral population dynamics, viral overdispersion across target cells would have important evolutionary consequences. First, reassortment between genetically and phenotypically distinct strains could bring together beneficial mutations on different gene segments or allow for a more effective purging of deleterious mutations. Second, viral overdispersion effectively produces ‘collective infectious units’ (Sanjuán 2017). The existence of these collective infectious units will put selection pressures on a virus to evolve cooperative traits, or, conversely, non-cooperative traits that would allow a virus to ‘cheat’. In either case, the importance of quantifying cellular MOI is clear, as MOI will determine the distribution of viral group sizes, which would in turn affect the types of ‘social interactions’ experienced by viral populations (Díaz-Muñoz, Sanjuán, and West 2017). The within-host macroparasite models presented here provide an approach for estimating the degree of viral overdispersion from fits to viral data. More generally, these models allow for the reality of cellular coinfection dynamics to be integrated into within-host disease models, in a scalable, low-dimensional fashion. While their general formulation has been developed here, these models require assumptions to be made between cellular input and various cellular phenotypes. Empirical studies examining the structure of these relationships is the next critical step to the continued development of these models, and towards their use in better understanding the within-host and evolutionary dynamics of viral infections.

## Supplementary Material

vez018_Supplementary_Data
