# Supplementary material for: Within-host infectious disease models accommodating cellular coinfection, with an application to influenza
Source: Virus Evol. 2019 Jul 8;5(2):vez018. doi: 10.1093/ve/vez018 (PMC6613536; doi:10.1093/ve/vez018)
Supplement: vez018_Supplementary_Data [file vez018_supplementary_data.pdf]

Supplementary Material

Contents

Supplementary Text S1:
Epidemiological macroparasite models **2**

Supplementary Text S2:
Negative binomial distribution for multiply infected cells **4**

Supplementary Text S3:
Statistical estimation of model parameters **18**

Supplementary Table S1:
Parameter estimates for the target cell limited microparasite model **20**

Supplementary Text S4:
Identifiability analysis **21**

Supplementary Figure S6:
Dynamics of the macroparasite model incorporating the innate immune response **27**

Supplementary References **28**

Supplementary Text S1: Epidemiological macroparasite models

The basic structure of epidemiological macroparasite models generally consists of only three variables [1]. The first variable is the total number of hosts H, the second is the total number of macroparasites P (e.g., worms) across those hosts, and the third is the total number of free-living parasites L. The rate of change in the number of hosts is generally given by:

$$\frac{dH}{dt} = aH - bH - H\sum_{i=0}^{\infty}\alpha_i p_i \tag{S1}$$

where a is the per capita birth rate of hosts and b is the background mortality of hosts. In this formulation, the host population grows exponentially in the absence of infection, provided that $a > b$. The term $-H\sum_{i=0}^{\infty}\alpha_i p_i$ captures disease-induced mortality, with α_i denoting the death rate of hosts infected with i macroparasites, and p_i denoting the fraction of the host population infected with i macroparasites. The rate of change in the number of free-living parasites is generally given by:

$$\frac{dL}{dt} = \left(H\sum_{i=0}^{\infty}\lambda_i p_i\right) - \eta L - \beta HL \tag{S2}$$

where the term $H\sum_{i=0}^{\infty}\lambda_i p_i$ represents the total rate at which infected hosts produce new free-living parasites, with λ_i being the rate at which hosts infected with i parasites produce free-living parasites. The term $-\eta L$ reflects natural loss of free-living parasites, and the term $-\beta HL$ reflects loss of free-living parasites due to entry into hosts.
The rate of change in the number of macroparasites in hosts is generally given by:

$$\frac{dP}{dt} = \beta HL - bH\sum_{i=0}^{\infty} ip_i - \mu P - H\sum_{i=0}^{\infty} i\alpha_i p_i \tag{S3}$$

where the term βHL reflects an increase in the number of macroparasites within hosts due to entry of free-living parasites into hosts, the term $-bH\sum_{i=0}^{\infty} ip_i$ captures macroparasite death by the death of hosts via factors unrelated to parasitism, and the term $-\mu P$ captures macroparasite death by natural death of the parasite within a host. The last term, $-H\sum_{i=0}^{\infty} i\alpha_i p_i$, captures the decrease in the number of macroparasites due to the death of the host that they themselves induced.

These three equations are traditionally simplified using a number of assumptions. The first assumption is that the rate at which free-living stages of parasite are produced from hosts is linearly dependent on the number of parasites in that host: $\lambda_i = \lambda i$, where λ is a scalar constant. This leads to simplification of the term $H\sum_{i=0}^{\infty}\lambda ip_i$ to $H\lambda\sum_{i=0}^{\infty} ip_i$, which can be further simplified to λP, given that $\sum_{i=0}^{\infty} ip_i$ is the mean number of parasites per host, i.e.$\frac{P}{H}$. The realization that $\sum_{i=0}^{\infty} ip_i = \frac{P}{H}$ also simplifies $-bH\sum_{i=0}^{\infty} ip_i$ to $-bP$. The second assumption is that the rate of disease-induced mortality scales linearly with macroparasite

burden: $\alpha_i = \alpha i$, where α is a scalar constant. This leads to simplification of the term $H\sum_{i=0}^{\infty}\alpha_i p_i$ to $\alpha H\sum_{i=0}^{\infty} i p_i$, which can be further simplified to αP. This also leads to simplification of the term $H\sum_{i=0}^{\infty} i\alpha_i p_i$ to $\alpha H\sum_{i=0}^{\infty} i^2 p_i$, which can be written as $\alpha H E(i^2)$, where $E(i^2)$ is the mean-square number of parasites per host. A final assumption is that $E(i^2)$ is a time-independent function of the mean number of parasites per host $\frac{P}{H}$. Most commonly in this literature, this function is given by a negative binomial distribution with mean $m = \frac{P}{H}$ and dispersion parameter k: $E(i^2) = m + m^2(1+k)/k$ [2], with empirical support for this negative binomial distribution provided in [3]. With these assumptions, equations (S1-S3) become:

$$\frac{dH}{dt} = aH - bH - \alpha P \tag{S4}$$

$$\frac{dL}{dt} = \lambda P - \eta L - \beta H L \tag{S5}$$

$$\frac{dP}{dt} = \beta H L - (b+\mu+\alpha)P - \frac{\alpha(i+k)}{k}\frac{P^2}{H} \tag{S6}$$

Frequently, an assumption is made that the characteristic time in the free living stage is short relative to that in the host, which leads to: $\frac{dL}{dt} \approx 0$. Solving equation (S5) for L yields: $L = \frac{\lambda P}{\eta + \beta H}$. Substituting this into equation (S6) yields:

$$\frac{dP}{dt} = \lambda P\frac{H}{\xi + H} - (b+\mu+\alpha)P - \frac{\alpha(1+k)}{k}\frac{P^2}{H} \tag{S7}$$

where the transmission efficiency constant $\xi = \frac{\eta}{\beta}$, and $\frac{H}{\xi+H}$ represents the probability that a free-living parasite successfully enters a host and becomes an adult parasite. Equations (S4-S6), or alternatively, equations (S4) and (S7) are classically used to model the population dynamics of macroparasites and their hosts.

Supplementary Text S2: Negative binomial distribution for multiply infected cells

In the main text, our target cell limited model does not explicitly incorporate an eclipse phase for cells. Eclipse phases for infected cells are often times explicitly included in within-host influenza models. They capture a waiting period between when a cell becomes infected and when it starts producing viral output. For influenza, the eclipse phase is known to last approximately 2-4 hours [2]. It is during the eclipse phase that cells are still allowing entry of additional virions; following the eclipse phase, often times superinfection exclusion occurs, with no additional entry of virus possible. Here, we formulate a basic within-host target cell limited model to explicitly include an eclipse phase. This formulation assumes that virions can enter cells only while cells are either uninfected or during their eclipse phase. Once they become productively infected, additional virions can no longer enter cells. Instead of considering a single compartment for cells in the eclipse phase, we consider multiple classes $\mathbb{E}_{i,j}$, where i denotes the current multiplicity of infection of a cell in this eclipse compartment and j in $1...m$ denotes the eclipse stage. The total number of eclipse stages m determines the distribution of time a cell remains in the eclipse phase. When $m = 1$, cells remain in the eclipse phase for an exponentially distributed amount of time. As m increases, the variance in the distribution of time a cell remains in the eclipse phase decreases. Under this formulation, the distribution of time a cell remains in the eclipse phase is gamma-distributed. Here, we will show that the distribution of cellular multiplicity of infection can be approximated by a negative binomial distribution when infected cells go through an eclipse phase, with the dispersion parameter of the negative binomial distribution determined by the number of eclipse stages m. We show this by first demonstrating that the initial distribution of cellular MOI is close to a negative binomial distribution. We then show that if solutions are negative binomially distributed initially, then they will remain negative binomially distributed throughout the initial infection period and after the target cell population has been depleted.

Our model is given by:

$$\frac{d}{dt}T = -\beta TV,$$

$$\frac{d}{dt}E_{1,1} = \beta TV - \beta E_{1,1}V - kE_{1,1},$$

$$\vdots$$

$$\frac{d}{dt}E_{i,1} = \beta E_{i-1,1}V - \beta E_{i,1}V - kE_{i,1}, \qquad i > 1$$

$$\vdots$$

$$\frac{d}{dt}E_{1,j} = kE_{1,j-1} - \beta E_{1,j}V - kE_{1,j}, \qquad 1 < j \leq m$$

$$\vdots$$

$$\frac{d}{dt}E_{i,j} = kE_{i,j-1} + \beta E_{i-1,j}V - \beta E_{i,j}V - kE_{i,j}, \qquad 1 < j \leq m,\ i > 1$$

$$\frac{d}{dt}I_i = kE_{i,m} - \delta I_i,$$

$$\frac{d}{dt}V = \sum_{i=1}^{\infty} p_i I_i - cV.$$

Here, T is the number of uninfected (and susceptible) target cells, $E_{i,j}$ is the number of eclipse phase cells that have internalized i viral particles and are in eclipse stage j (out of m), I_i is the number of productively infected cells with i internalized particles, and V is the amount of free virus.

The Multiplicity of Infection is Nearly Negative Binomially Distributed Initially

To show that the initial distribution is close to a negative binomial distribution, we first find an approximate solution to the viral load, then approximate the solutions to the remaining variables of the system.

Approximation of Solutions for Virus Concentration $V(t)$

We first consider the initial infection period where the target cell population is approximately constant, i.e.

$$T(t) \approx T(0) =: T_0 \tag{S8}$$

Initially, viral growth is driven by the production of viruses from singly infected cells, which allows us to make the approximation:

$$\sum_{i=1}^{\infty} p_i I_i \approx p_1 I_1. \tag{S9}$$

During the initial infection, it is reasonable to assume that that coinfection is infrequent, i.e.:

$$k >> \beta V(t). \tag{S10}$$

This gives an approximation of the differential equations of singly infected eclipse phase cell populations, which we can solve analytically.

$$\frac{d}{dt}E_{1,1} = \beta TV - \beta E_{1,1}V - kE_{1,1} \approx \beta T_0 V - kE_{1,1},$$

and

$$\frac{d}{dt}E_{1,j} = \beta E_{1,j-1}V - \beta E_{1,j}V - kE_{1,j} \approx \beta V E_{1,j-1} - kE_{1,j}.$$

Combining the above equations and using the assumption in equation (S9), we form the following ODE system for singly infected cells:

$$
\begin{aligned}
&\frac{d}{dt}E_{1,1} \approx \beta T_0 V - kE_{1,1},\\
&\frac{d}{dt}E_{1,2} \approx kE_{1,1} - kE_{1,2},\\
&\vdots\\
&\frac{d}{dt}E_{1,j} \approx kE_{1,j-1} - kE_{1,j}, \qquad 1 < j \leq m\\
&\frac{d}{dt}I_1 = kE_{1,m} - \delta I_1,\\
&\frac{d}{dt}V \approx p_1 I_1 - cV.
\end{aligned}
$$

We note that this system of equations is linear, so solutions will be a sum of exponential terms. Thus, we have the following approximation for virus concentration:

$$V(t) \approx V_0 e^{\lambda t}. \tag{S11}$$

We note that $V_0 \neq V(0)$, as the above estimate does not include the initial viral loss that occurs as the infection becomes established. Thus, V_0 is what the initial viral condition would be if exponential viral growth occurred immediately. Due to this, we know $V(0) > V_0$.

Approximations of Solutions for Infected Cells $I_i(t)$

Using assumptions (S8) and (S10), and approximation (S11), we have the following system of equations, which we will solve:

$$
\begin{aligned}
&T(t) = T_0,\\
&\frac{d}{dt}E_{1,1} = \beta T_0 V_0 e^{\lambda t} V - kE_{1,1}, && \text{(S12)}\\
&\quad\vdots\\
&\frac{d}{dt}E_{i,1} = \beta E_{i-1,1} V_0 e^{\lambda t} - kE_{i,1}, \qquad i > 1 && \text{(S13)}\\
&\quad\vdots\\
&\frac{d}{dt}E_{1,j} = kE_{1,j-1} - kE_{1,j}, \qquad 1 < j \leq m && \text{(S14)}\\
&\quad\vdots\\
&\frac{d}{dt}E_{i,j} = kE_{i,j-1} + \beta E_{i-1,j} V_0 e^{\lambda t} - kE_{i,j}, \qquad 1 < j \leq m,\ i > 1 && \text{(S15)}\\
&\frac{d}{dt}I_i = kE_{i,m} - \delta I_i. && \text{(S16)}
\end{aligned}
$$

We begin by solving equation (S12) for $E_{1,1}$, which has solution

$$E_{1,1}(t) = \frac{T_0\beta V_0}{k+\lambda}e^{\lambda t} - \frac{T_0\beta V_0}{k+\lambda}e^{-kt}.$$

We approximate this by the leading exponential term:

$$E_{1,1}(t) \approx \frac{T_0\beta V_0}{k+\lambda}e^{\lambda t}.$$

We now aim to solve equation (S13) for all $E_{i,1}$. Using this approximation, we can solve for $E_{2,1}$, which has differential equation

$$\begin{aligned}\frac{d}{dt}E_{2,1} &\approx \beta E_1 V_0 e^{\lambda t} - kE_{2,1}\\ &\approx \beta\Big[\frac{T_0\beta V_0}{k+\lambda}e^{\lambda t}\Big]V_0 e^{\lambda t} - kE_{2,1}\\ &\approx \frac{T_0\beta^2 V_0^2}{k+\lambda}e^{2\lambda t} - kE_{2,1},\end{aligned}$$

which has solution

$$E_{2,1}(t) = \frac{T_0\beta^2 V_0^2}{(k+\lambda)(k+2\lambda)}e^{\lambda t} - \frac{T_0\beta V_0}{(k+\lambda)(k+2\lambda)}e^{-kt} \approx \frac{T_0\beta^2 V_0^2}{(k+\lambda)(k+2\lambda)}e^{\lambda t}.$$

Note that our approximations of $E_{1,1}$ and $E_{2,1}$ follows the patern $E_{i,1}(t) = \frac{T_0(\beta V_0)^i}{\prod_{n=1}^{i}(k+n\lambda)}e^{i\lambda t}$. Continuing to solve equation (S13) in this manner shows

$$\begin{aligned}E'_{i,1} &\approx \beta E_{1,i-1}V_0 e^{\lambda t} - kE_{i,1}\\ &\approx \beta\frac{T_0(\beta V_0)^{i-1}}{\prod_{n=1}^{i-1}(k+n\lambda)}e^{(i-1)\lambda t}V_0 e^{\lambda t} - kE_{i,1}\\ &\approx \frac{T_0(\beta V_0)^i}{\prod_{n=1}^{i-1}(k+n\lambda)}e^{i\lambda t} - kE_{i,1},\end{aligned}$$

which has approximate solution

$$E_{i,1}(t) \approx \frac{T_0(\beta V_0)^i}{\prod_{n=1}^{i}(k+n\lambda)}e^{i\lambda t}.$$

Thus, all $E_{i,1}$ follow this pattern.
We now move on to solving equation (S14). We begin by solving for $E_{1,2}$, which has differential equation

$$\frac{d}{dt}E_{1,2} \approx kE_{1,1} - kE_{1,2} \approx k\frac{T_0\beta V_0}{k+\lambda}e^{\lambda t} - kE_{1,2}$$

which has approximate solution

$$E_{1,2}(t) \approx \frac{T_0\beta V_0 k}{(k+\lambda)^2}e^{\lambda t}$$

Note that our approximations of $E_{1,1}$ and $E_{1,2}$ follows the pattern $E_{1,j}(t) = \dfrac{T_0\beta V_0 k^{j-1}}{(k+\lambda)^j}e^{\lambda t}$. Continuing to solve equation (S14) in this manner shows

$$\begin{aligned}\frac{d}{dt}E_{1,j} &\approx kE_{1,j-1} - kE_{1,j}\\ &\approx k\frac{T_0\beta V_0 k^{j-2}}{(k+\lambda)^{j-1}}e^{\lambda t} - kE_{1,j}\end{aligned}$$

which has approximate solution

$$E_{1,j}(t) \approx \frac{T_0\beta V_0 k^{j-1}}{(k+\lambda)^j}e^{\lambda t}.$$

Thus all $E_{1,j}$ follow this pattern.

For ease of notation, we define $p_n = \dfrac{1}{k+n\lambda}$, allowing us to write equations () and () as:

$$\begin{aligned}E_{i,1}(t) &\approx T_0(\beta V_0)^i e^{i\lambda t}\prod_{n=1}^{i} p_n,\\ E_{1,j}(t) &\approx T_0\beta V_0 k^{j-1}e^{\lambda t}p_1^j.\end{aligned}$$

Thus, using equation (S15), the differential equation for $E_{2,2}$ is

$$\begin{aligned}\frac{d}{dt}E_{2,2} &\approx kE_{2,1} + \beta E_{1,2}V - kE_{2,2}\\ &\approx kT_0(\beta V_0)^2 e^{2\lambda t}p_1p_2 + \beta T_0\Big[\beta V_0 k e^{\lambda t}p_1^2\Big]V_0e^{\lambda t} - kE_{2,2}\\ &\approx T_0(\beta V_0)^2 k e^{2\lambda t}\Big(p_2 + p_1\Big)p_1 - kE_{2,2}.\end{aligned}$$

The approximate solution to this is

$$E_{2,2}(t) \approx T_0(\beta V_0)^2 k e^{2\lambda t}\Big(p_2 + p_1\Big)p_1 \cdot \frac{1}{k+2\lambda} = T_0(\beta V_0)^2 k e^{2\lambda t}\Big(p_2 + p_1\Big)p_1p_2.$$

Then, the differential equation for $E_{2,3}$ is

$$\begin{aligned}\frac{d}{dt}E_{2,3} &\approx kE_{2,2} + \beta E_{1,3}V - kE_{2,3}\\ &\approx kT_0(\beta V_0)^2 k e^{2\lambda t}\Big(p_2 + p_1\Big)p_1p_2 + \beta T_0\Big[\beta V_0 k^2 e^{\lambda t}p_1^3\Big]V_0e^{\lambda t} - kE_{2,3}\\ &\approx T_0(\beta V_0)^2 k^2 e^{2\lambda t}\Big(p_1^2 + p_1p_2 + p_2^2\Big)p_1 - kE_{2,2}.\end{aligned}$$

The approximate solution to this is

$$\begin{aligned}E_{2,3}(t) &\approx T_0(\beta V_0)^2 k^2 e^{2\lambda t}\Big(p_1^2 + p_1p_2 + p_2^2\Big)p_1 \cdot \frac{1}{k+2\lambda}\\ &= T_0(\beta V_0)^2 k e^{2\lambda t}\Big(p_1^2 + p_1p_2 + p_2^2\Big)p_1p_2.\end{aligned}$$

Notice that the terms inside the parentheses are the polynomials of degree 2 (more generally j-1) of the set $\{p_1, p_2\}$ (more generally $\{p_1, ..., p_i\}$). We test this for $E_{3,2}$ then continue with general i and j.

The differential equation for $E_{3,2}$ is

$$\begin{aligned}\frac{d}{dt}E_{3,2} &\approx kE_{3,1} + \beta E_{2,2}V - kE_{3,2} \\ &\approx kT_0(\beta V_0)^3 e^{3\lambda t} p_1 p_2 p_3 + \beta\Big[T_0(\beta V_0)^2 k e^{2\lambda t}\Big(p_2 + p_1\Big)p_1 p_2\Big]V_0 e^{\lambda t} - kE_{3,2} \\ &\approx T_0(\beta V_0)^3 k e^{3\lambda t}\Big(p_1 + p_2 + p_3\Big)p_1 p_2 - kE_{2,2}.\end{aligned}$$

The approximate solution to this is

$$\begin{aligned}E_{3,2}(t) &\approx T_0(\beta V_0)^3 k e^{3\lambda t}\Big(p_1 + p_2 + p_3\Big)p_1 p_2 \cdot \frac{1}{k + 3\lambda} \\ &= T_0(\beta V_0)^3 k e^{3\lambda t}\Big(p_1 + p_2 + p_3\Big)p_1 p_2 p_3.\end{aligned}$$

We note that all of the above terms in this section are described by:

$$E_{i,j}(t) \approx T_0 \left(V_0 \beta\right)^i k^{j-1} e^{i\lambda t} \sum_{\dagger_{j-1}} p_1^{\alpha_1} p_2^{\alpha_2} ... p_i^{\alpha_i} \prod_{n=1}^{i} p_n,$$

where $\sum_{\dagger_{j-1}} p_1^{\alpha_1} p_2^{\alpha_2} ... p_i^{\alpha_i}$ is all combinations of degree $j-1$ of the set $\{p_1, p_2, ..., p_i\}$ We now proceed to find the approximate solution for general i and j.

$$\begin{aligned}\frac{d}{dt}E_{i,j} &\approx kE_{i,j-1} + \beta E_{i-1,j} V_0 e^{\lambda t} - kE_{i,j} \\ &\approx k\Big[T_0 \left(V_0\beta\right)^i k^{j-2} e^{i\lambda t} \sum_{\dagger_{j-2}v} p_1^{\alpha_1} p_2^{\alpha_2} ... p_i^{\alpha_i} \prod_{n=1}^{i} p_n\Big] \\ &\quad + \beta\Big[T_0 \left(V_0\beta\right)^{i-1} k^{j-1} e^{(i-1)\lambda t} \sum_{\dagger_{j-1}} p_1^{\alpha_1} p_2^{\alpha_2} ... p_{i-1}^{\alpha_{i-1}} \prod_{n=1}^{i-1} p_n\Big] V_0 e^{\lambda t} - kE_{i,j} \\ &\approx T_0 \left(V_0\beta\right)^i k^{j-1} e^{i\lambda t} \prod_{n=1}^{i-1} p_n \Big[p_i \sum_{\dagger_{j-2}} p_1^{\alpha_1} p_2^{\alpha_2} ... p_i^{\alpha_i} + \sum_{\dagger_{j-1}} p_1^{\alpha_1} p_2^{\alpha_2} ... p_{i-1}^{\alpha_{i-1}}\Big] - kE_{i,j}.\end{aligned}$$

In order for us to obtain the desired result, we must show that

$$p_i \sum_{\dagger_{j-2}} p_1^{\alpha_1} p_2^{\alpha_2} ... p_i^{\alpha_i} + \sum_{\dagger_{j-1}} p_1^{\alpha_1} p_2^{\alpha_2} ... p_{i-1}^{\alpha_{i-1}} = \sum_{\dagger_{j-1}} p_1^{\alpha_1} p_2^{\alpha_2} ... p_i^{\alpha_i}.$$

This can be seen by considering how to deconstruct all the combinations of degree $j-1$ of the set $\{p_1, p_2, ..., p_i\}$ into the pieces with p_i and without p_i. All terms without p_i are described by $\sum_{\dagger_{j-1}} p_1^{\alpha_1} p_2^{\alpha_2} ... p_{i-1}^{\alpha_{i-1}}$. The remaining are all multiplied by p_i; factoring it out

leaves all possible combinations of degree $j-2$, i.e. $\sum_{\dagger_{j-2}} p_1^{\alpha_1} p_2^{\alpha_2} ... p_i^{\alpha_i}$. Thus, the desired equality holds. Solving this differential equation provides

$$E_{i,j} \approx T_0 \left(V_0 \beta\right)^i k^{j-1} e^{i\lambda t} \prod_{n=1}^{i-1} p_n \Big[\sum_{\dagger_{j-1}} p_1^{\alpha_1} p_2^{\alpha_2} ... p_i^{\alpha_i} \Big] \frac{1}{k + i\lambda}$$

which simplifies to

$$E_{i,j} \approx T_0 \left(V_0 \beta\right)^i k^{j-1} e^{i\lambda t} \prod_{n=1}^{i} p_n \Big[\sum_{\dagger_{j-1}} p_1^{\alpha_1} p_2^{\alpha_2} ... p_i^{\alpha_i} \Big]$$

as our solution to (S15). With this solution, we can solve equation (S16) to gain the following approximation for I_i:

$$I_i(t) \approx \frac{T_0 \left(V_0 \beta\right)^i k^m \sum_{\dagger_{m-1}} p_1^{\alpha_1} p_2^{\alpha_2} ... p_i^{\alpha_i}}{(i\lambda + \delta) \prod_{n=1}^{i} (k + n\lambda)} e^{i\lambda t}, \qquad p_n = \frac{1}{k + n\lambda}.$$

${}^{\dagger_{m-1}} \alpha_i \in \mathbb{Z}, \;\; \alpha_i \geq 0, \;\; \sum_{j=1}^{i} \alpha_j = m - 1$, where m is the number of eclipse stages.

Negative Binomial Distribution of I_i

Note that $p_n \leq p_1$ for all $n \geq 1$. Thus, we have the following:

$$\begin{aligned} I_i(t) &\approx \frac{T_0 \left(V_0 \beta\right)^i k^m \sum_{\dagger_{m-1}} p_1^{\alpha_1} p_2^{\alpha_2} ... p_i^{\alpha_i}}{(i\lambda + \delta) \prod_{n=1}^{i} (k + n\lambda)} e^{i\lambda t} \\ &\leq \frac{T_0 \left(V_0 \beta\right)^i k^m \sum_{\dagger_{m-1}} p_1^{\alpha_1} p_1^{\alpha_2} ... p_1^{\alpha_i}}{(\lambda + \delta) \prod_{n=1}^{i} (k + \lambda)} e^{i\lambda t} \\ &= \frac{T_0 \left(V_0 \beta\right)^i k^m \sum_{\dagger_{m-1}} p_1^{\alpha_1 + \alpha_2 + ... \alpha_i}}{(\lambda + \delta)(k + \lambda)^i} e^{i\lambda t} \\ &= \frac{T_0 k^m}{(\lambda + \delta)} \sum_{\dagger_{m-1}} p_1^{\alpha_1 + \alpha_2 + ... \alpha_i} \Big(\frac{V_0 \beta}{k + \lambda} \Big)^i e^{i\lambda t}. \end{aligned}$$

We wish to show

$$\sum_{\dagger_{m-1}} p_1^{\alpha_1 + \alpha_2 + ... \alpha_i} = \binom{i + m - 2}{m - 1} p_1^{m-1}.$$

This can be seen by considering how many ways we can choose i integers $\alpha_i \geq 0$ such that they add up to $m-1$. If we are on a lattice at (1,1) and heading toward the point (i, m), we can increase α_1 by one integer value by moving one step in the direction (0,1) or change to

α_2 by moving one step in the direction (1,0). Thus there will be $(i-1)+(m-1) = i+m-2$ total moves, and we must choose the position of the $m-1$ of them that will be in the direction (0,1).

Thus, we have

$$I_i(t) \leq \frac{T_0 k^m}{(\lambda+\delta)(k+\lambda)^{m-1}} \binom{i+m-2}{m-1} \Big(\frac{V_0\beta}{k+\lambda}\Big)^i e^{i\lambda t}.$$

If we define

$$\alpha = \frac{V_0\beta T_0}{(\lambda+\delta)} \Big(\frac{k}{k+\lambda-V_0\beta}\Big)^m, \tag{S17}$$

Then at $t=0$, we can rewrite our upper bound for I_i to be

$$I_i(0) \leq \alpha \binom{i+m-2}{i-1} \Big(\frac{k+\lambda-V_0\beta}{k+\lambda}\Big)^m \Big(\frac{V_0\beta}{k+\lambda}\Big)^{i-1}, \tag{S18}$$

which is α times a negative binomial probability mass function with dispersion parameter m and probability of success $\frac{V_0\beta}{k+\lambda}$. Note that the our number of successes is i, with support $\{1,2,3,...\}$. Further, our assumption in equation (S10), which is assumed for the initial infection process, implies $k >> \beta V(0)$, and thus $\frac{V_0\beta}{k+\lambda} < \frac{k}{k+\lambda} < 1$ is truly a probability.

We note that this upper bound is equal to the approximation for I_1. Further, we note that the distance between the upper bound and the actual value increases with i. Thus, at least for small values of i, we expect that this upper bound could be a reasonable approximation for I_i.

A Negative Binomial Distribution is Maintained During the Initial Infection and After Target Cell Depletion

During the Initial Infection

Suppose that our solutions to I_i have the form

$$I_i(t) = \gamma(t) \binom{i+m-2}{i-1} (1-\phi)^m \phi^{i-1} e^{(i-1)\lambda t},$$

for some $\gamma(t) > 0$ and $\phi \in (0,1)$. We restrict our consideration to $t \in \Big[0, \frac{-1}{\lambda}\log(\phi)\Big)$. This implies $\phi e^{\lambda t} < 1$ for all t we consider. In the calculations below, we use the following identity:

$$\frac{1}{(1-\phi)^m} = \sum_{i=1}^{\infty} \binom{i+m-2}{i-1} \phi^{i-1},$$

with $0 < \phi < 1$.

Thus we see

$$\sum_{i=1}^{\infty} I_i(t) = \sum_{i=1}^{\infty} \gamma(t) \binom{i+m-2}{i-1} (1-\phi)^m \phi^{i-1} e^{(i-1)\lambda t}$$

$$
\begin{aligned}
&= \gamma(t)(1-\phi)^m \sum_{i=1}^{\infty} \binom{i+m-2}{i-1} (\phi e^{\lambda t})^{i-1} \\
&= \gamma(t)(1-\phi)^m \frac{1}{(1-\phi e^{\lambda t})^m} \\
&= \gamma(t)\Big(\frac{1-\phi}{1-\phi e^{\lambda t}}\Big)^m.
\end{aligned}
$$

Thus, at any time t the proportion of cells infected with i virions is

$$
\begin{aligned}
\frac{I_i(t)}{\sum_{n=1}^{\infty} I_n(t)} &= \frac{\gamma(t)\binom{i+m-2}{i-1}(1-\phi)^{m-1}\phi^{i-1}e^{(i-1)\lambda t}}{\gamma(t)\Big(\frac{1-\phi}{1-\phi e^{\lambda t}}\Big)^m} \\
&= \binom{i+m-2}{i-1}(1-\phi)^m \phi^{i-1} e^{(i-1)\lambda t}\Big(\frac{1-\phi e^{\lambda t}}{1-\phi}\Big)^m \\
&= \binom{i+m-2}{i-1}\Big(1-\phi e^{\lambda t}\Big)^m \Big(\phi e^{\lambda t}\Big)^{i-1},
\end{aligned}
$$

which is binomially distributed with parameter $pe^{\lambda t}$. Since we restrict our considerations to the timeframe $t \in \Big[0, \frac{-1}{\lambda}\log(p)\Big)$, we know that $\phi e^{\lambda t} < 1$.

Combining the results above with equation (S18), we see that we approximately have a negative binomial distribution for $t \in \Big[0, \frac{-1}{\lambda}\log(\phi)\Big)$, with dispersion parameter m, and probability $\phi = \frac{V_0\beta}{k+\lambda}$, giving us the following negative binomial distribution:

$$
\frac{I_i(t)}{\sum_{n=1}^{\infty} I_n(t)} = \binom{i+m-2}{i-1}\Big(1-\frac{V_0\beta e^{\lambda t}}{k+\lambda}\Big)^m \Big(\frac{V_0\beta e^{\lambda t}}{k+\lambda}\Big)^{i-1}. \tag{S19}
$$

After the Target Cell Population is Depleted

To show that we will have a negative binomial distribution for later times, a similar argument as the above shows that if

$$
I_i(t) = \gamma(t)\binom{i+m-2}{i-1}(1-\phi)^m\phi^{i-1}, \tag{S20}
$$

then the solutions will maintain their binomial distribution (with the same dispersion parameter and probability) for all t.

Define t_D be the time at which target cells are depleted. Then, all of the infected virion producing cell populations have the following approximation:

$$
I_i(t) \approx I_i(t_D)e^{-\delta t}
$$

for $t \geq t_D$. If $I_i(t_D)$ is negatively binomially distributed, then $I_i(t)$ can be rewritten to match equation (S20), and thus will maintain its distribution, with the same dispersion parameter and probability, for all $t \geq t_D$.

Model Simulations Showing That I_i are Negative Binomially Distributed

We ran simulations to see if the infected cells follow a negative binomial distributions. We used the following parameters: $T_0 = 4 \times 10^8$ cells, $V(0)$=0.675 TCID_{50}/ml, $\beta = 3.375 \times 10^{-6}$ $(\text{TCID}_{50}/\text{ml})^{-1}\text{d}^{-1}$, $k = 120$ d^{-1}, $\delta = 5.2$ d^{-1}, $p = 0.46$ $\text{TCID}_{50}/\text{ml}\times\text{d}^{-1}$, $c = 15$ d^{-1}, and 15 eclipse phase stages. Our value for k may seem unreasonably high, however with 15 eclipse phase stages, the average time to maturation will be 3 hours, which agrees with the observations in [2]. Numerically, we cannot allow infinitely many infections, so we choose to allow 11 in total.

As we can see in the following figures, the distribution of infected cells is well approximated by a negative binomial distribution. For a selection of time points, we plot the frequencies of the first ten infected cell populations and the negative binomial distribution $\text{NB}(m, \phi)$ obtained by using $m = 15$ 'failures' (the number of eclipse phase stages) and probability $\phi = 1 - F_{I_1}^{1/m}$ of success, where F_{I_1} is the relative frequency of I_1 (i.e. the probability of no successful coinfections).

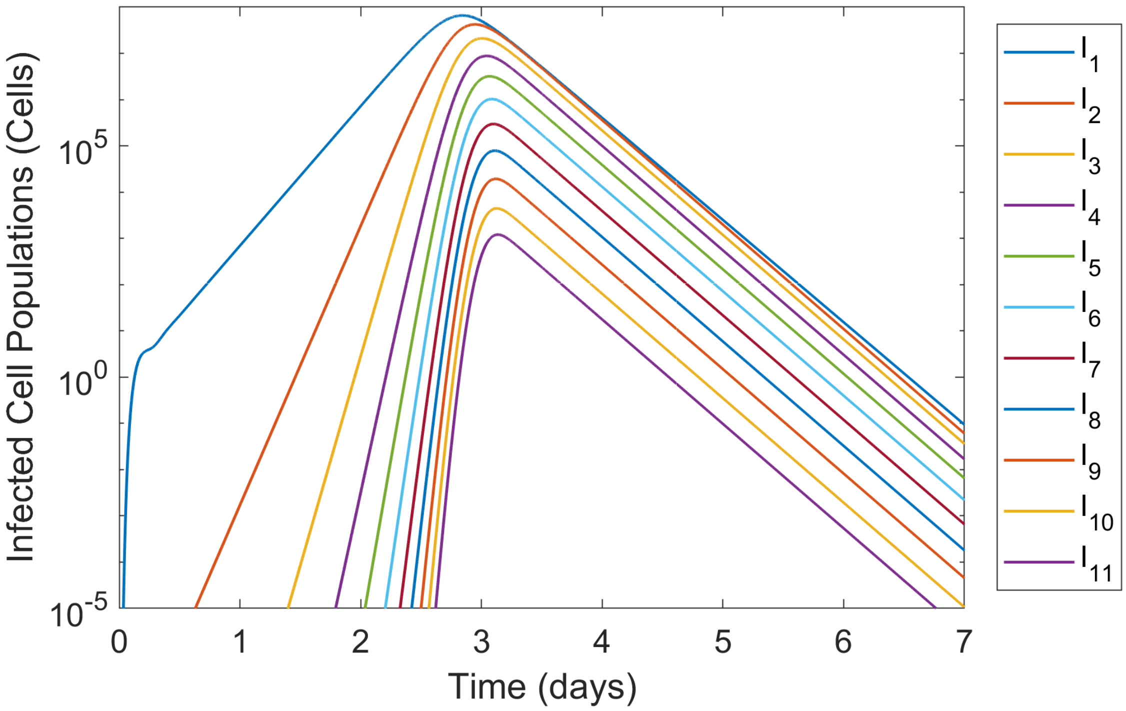

Figure 1: Model simulation showing infected virion producing cell populations using the following parameters: $T_0 = 4 \times 10^8$ cells, $V(0)$=0.675 TCID_{50}/ml, $\beta = 3.375 \times 10^{-6}$ $(\text{TCID}_{50}/\text{ml})^{-1}\text{d}^{-1}$, $k = 120$ d^{-1}, $\delta = 5.2$ d^{-1}, $p = 0.46$ $\text{TCID}_{50}/\text{ml}\times\text{d}^{-1}$, $c = 15$ d^{-1}, and 15 eclipse phase stages. I_i denotes virion producing cells infected with i virions.

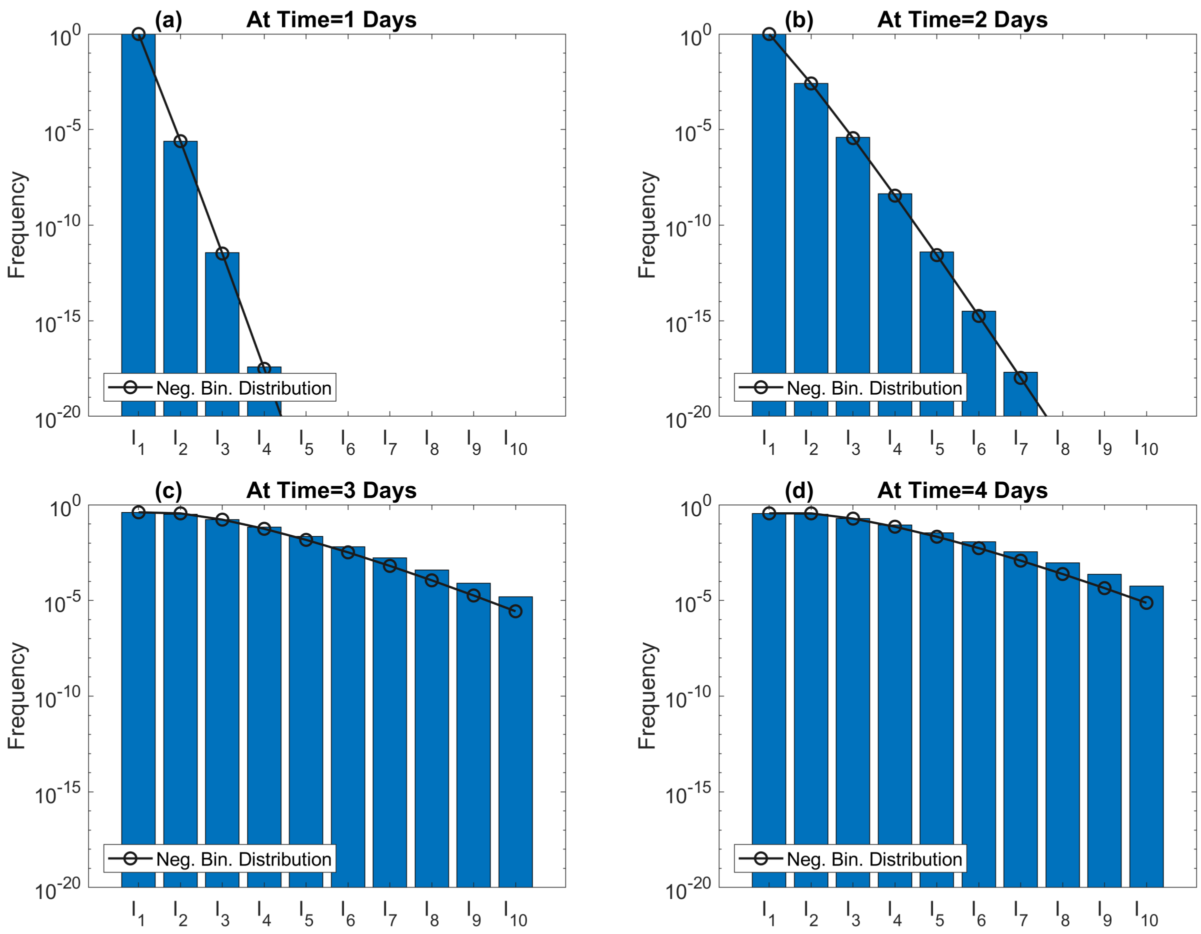

Figure 2: Relative frequencies of multiply infected virion producing cells from the simulation displayed in figure 1 at one day (a), two days (b), three days (c), and four days (d) post infection. I_i denotes virion producing cells infected with i virions. The plotted negative binomial distribution is NB(m, ϕ), where $m = 15$ is the number of 'failures' (number of eclipse phase stages) and ϕ is the probability of success. We use the frequency of I_1, which we denote F_{I_1} to find ϕ via the following equation: $\phi = 1 - F_{I_1}^{r^{-1}}$.

The above model simulations assume that the death rate of productively infected cells is a constant, given by δ. We can instead assume that the death rate scales linearly with the cellular multiplicity of infection i, such that $\delta_i = \delta_{constant} i$. Under this alternative form for the cellular death rate, the distribution of infected cells' multiplicity of infections remains distributed according to a negative binomial distribution. See figures (3) and (4) below.

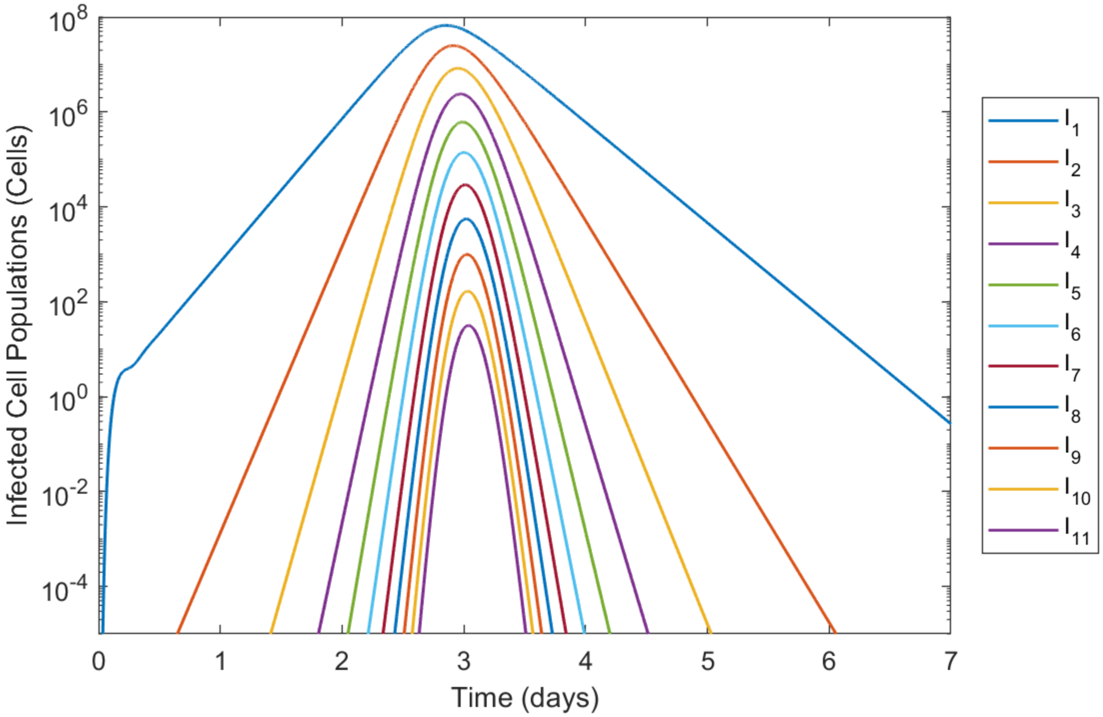

Figure 3: Model simulation showing infected virion producing cell populations using the following parameters: $T_0 = 4 \times 10^8$ cells, $V(0)$=0.675 TCID_{50}/ml, $\beta = 3.375 \times 10^{-6}$ $(\text{TCID}_{50}/\text{ml})^{-1}\text{d}^{-1}$, $k = 120$ d^{-1}, $\delta_{constant} = 5.2$ d^{-1}, $p = 0.46$ $\text{TCID}_{50}/\text{ml}\times\text{d}^{-1}$, $c = 15$ d^{-1}, and 15 eclipse phase stages. I_i denotes virion producing cells infected with i virions.

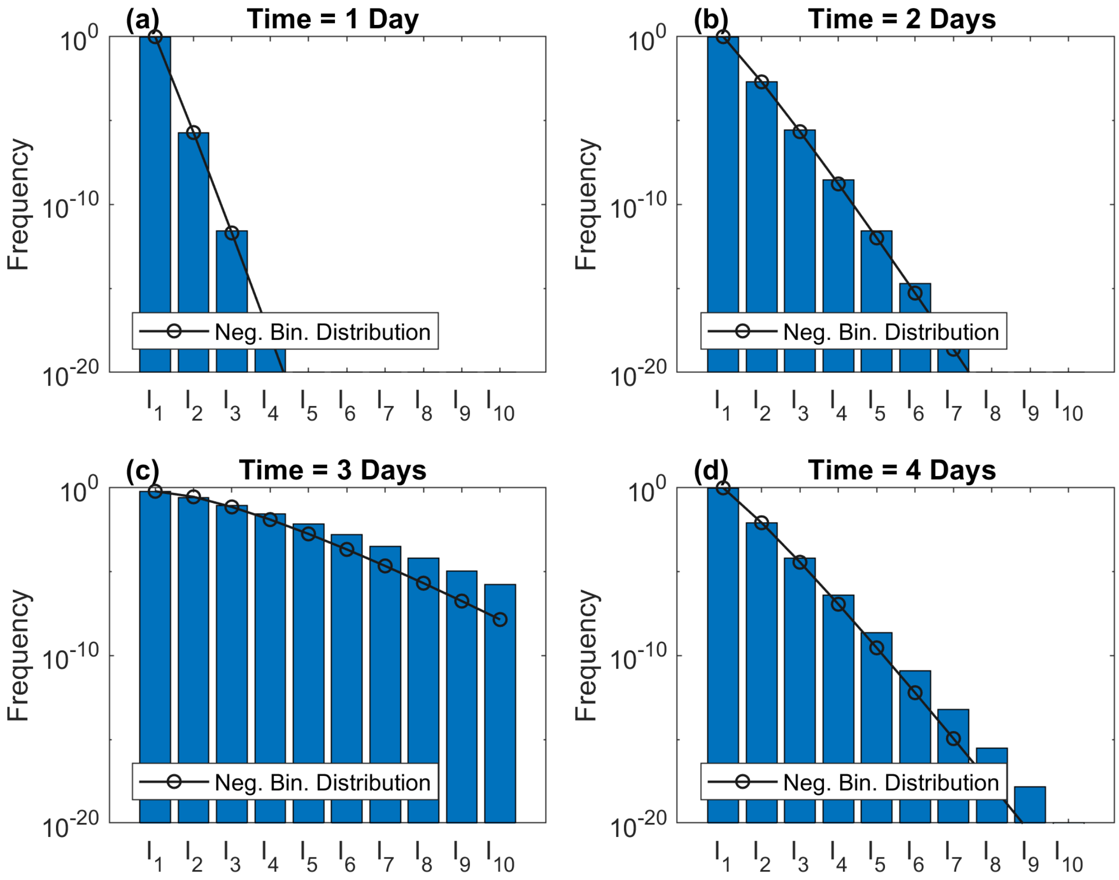

Figure 4: Relative frequencies of multiply infected virion producing cells from the simulation displayed in figure 3 at one day (a), two days (b), three days (c), and four days (d) post infection.

Direct Comparison of Model Simulations to the Analytical Findings

Using the same parameters as above, we plot the bar graphs from our simulation, but use the negative binomial distribution in equation (S19). We note that we only have simulations until day three. This is because our estimate only holds until $t = \frac{1}{\lambda}\log\left(\frac{k+\lambda}{V_0\beta}\right)$, is 3.1239 for this parameter set. Thus, we do not have estimates after this time. Further, we note that this estimate is valid during the initial growth period, when I_1 is assumed to be the largest contributer to virion production (equation (S9)). As seen in figure 1, $I_1 >> I_2$ does not hold at three days post infection. Combined with the fact that $p_i = p$ for our simulation, we can see that our estimate is no longer guaranteed to hold at day three, which is why we do not plot have a plot for that time.

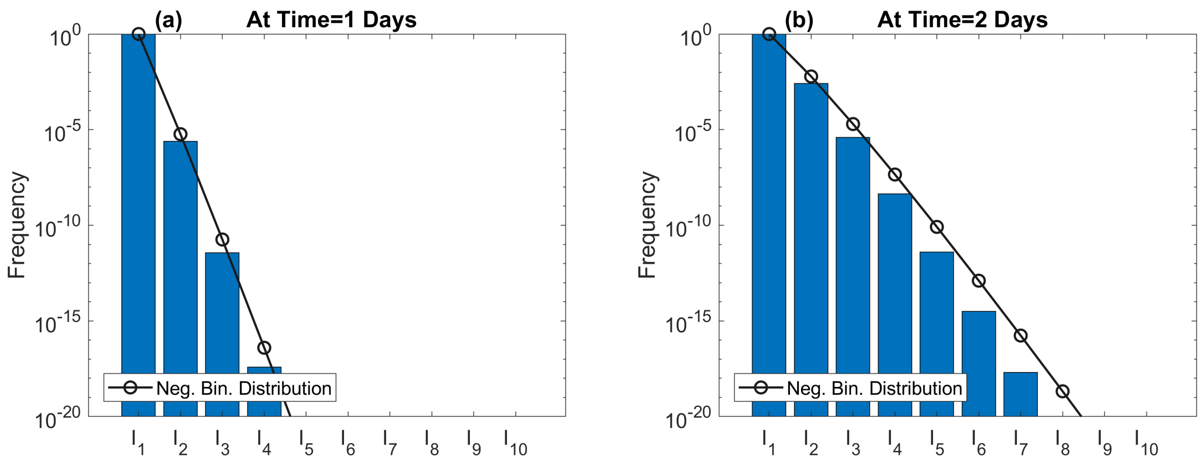

Figure 5: Relative frequencies of multiply infected virion producing cells from the simulation displayed in figure 1 at one day (a), and two days (b) post infection. I_i denotes virion producing cells infected with i virions. The plotted negative binomial distribution is $\mathrm{NB}(m, \phi)$, using the parameters from equation (S19): $m = 15$ is the number of 'failures' (eclipse phase stages) and $\phi = \dfrac{V_0 \beta e^{\lambda t}}{k + \lambda}$.

Supplementary Text S3: Statistical estimation of model parameters

We estimate model parameters for both of the target cell limited models and the macroparasite model incorporating the innate immune response using maximum likelihood. The general likelihood expression is already described in the literature, in an application to dengue virus within-host dynamics [3,4].

For the target-cell limited model, we assume $\log_{10}$ viral load measurements have normally-distributed measurement error, with standard deviation of $\sigma_E = 1$. The likelihood expression is given by:

$$\mathcal{L}(\gamma|D_j) = \prod_{k=1}^{n} [\theta(D_j(t_k)|M(\gamma, t_k), \sigma_E)\mathbb{1}_{D_j(t_k)>LOD} + \phi(LOD|M(\gamma, t_k), \sigma_E)\mathbb{1}_{D_j(t_k)\leq LOD}]$$

where the likelihood of the model γ, given pony j's viral load data D_j, depends on viral measurements that are both above and below the limit of detection (LOD). For each measurement that is above the limit of detection, the first of the two terms in the product expression applies. For each measurement that falls below the limit of detection, the second of the two terms applies. $D_j(t_k)$ specifies the viral load measurement (on the $\log_{10}$ scale) from pony j taken on day since infection k. θ is the Gaussian probability density function, evaluated at measurement $D_j(t_k)$. ϕ is the Gaussian cumulative distribution function, evaluated at the limit of detection.

For the macroparasite model incorporating the innate immune response, we fit the model to either two or three sets of data. When fitting the model to two sets of data, we use viral load measurements and interferon-α measurements (in units of fold change) as our data. When fitting the model to three sets of data, we additionally use the number of target cells remaining at the end of infection. Data are available for each pony for the first two of these datasets. For the number of remaining target cells, we use an estimate of 73% for each pony, based on an existing estimate in the literature that has been used in fitting of a microparasite version of this model to data [5]. To estimate model parameters, we first, for any given set of parameters γ, calculate the likelihood of each set of data independently. The likelihood expression for the viral load measurements is given above. The likelihood expression for the interferon measurements is given by:

$$\mathcal{L}(\gamma|F_j) = \prod_{k=1}^{n} \theta(F_j(t_k)|M(\gamma, k_k), \sigma_I)$$

where $M(\gamma, t_k)$ are model-predicted interferon fold change values, and we set $\sigma_I = 1$. The likelihood expression for the final number of target cells is given by:

$$\mathcal{L}(\gamma|T_j) = \theta\big((1 - 0.27)(3.5 \times 10^{11})|M(\gamma, t_{10}), \sigma_T\big)$$

where $M(\gamma, t_{10})$ are the model-predicted total number of target cells remaining at the end of the simulation and we set $\sigma_T = 1 \times 10^{11}$ cells.
We combine these dataset-specific likelihood values by taking the logs of each one and then summing them. When considering only viral load measurements and interferon fold change measurements, we sum only the first two of the log-likelihood values.

Supplementary Table S1: Parameter estimates for the target cell limited microparasite model

Maximum likelihood parameter estimates for the classic target cell limited microparasite model.

			PONY					
			1	2	3	4	5	6
**Parameter or initial condition**	**Units**	**Estimated or set**	**Value**					
T(0)	cells	Set	3.50E+11					
I(0)	cells	Set	0					
$\log_{10}(V(0))$	$\log_{10}$ RNA copies/ml NS	Estimated (individually)	-3.84	-22.72	-23.07	-19.34	-7.13	-20.21
λ	RNA copies/ml NS/day	Estimated (by group)	1.73E-06					
α	day^{-1}	Estimated (by group)	1.42					
η	day^{-1}	Estimated (individually)	8.43	1.03	12.72	1.35	8.45	18.98
β	$(RNA\ copy)^{-1}$ ml NS day^{-1}	Estimated (individually)	6.87E-04	1.50E-02	7.34E-03	5.51E-03	1.45E-03	6.03E-03

Supplementary Text S4: Identifiability analysis

Identifiability of the target-cell limited macroparasite model

Our within-host macroparasite model is given by equations (4), (5), and (7) in the main text, reproduced here:

$$\frac{dH}{dt} = -\alpha P,$$
$$\frac{dV}{dt} = \lambda P - \eta V - \beta HV,$$
$$\frac{dP}{dt} = \beta HV - \alpha P - \alpha\gamma\frac{P^2}{H},$$

where γ is a constant given by $(1+k)/k$. Here, we ask whether all parameters of the model are structurally identifiable when our observations are those of free virus:

$$y = V.$$

Changing notation of a derivative from primes to dots (to allow for easier notation of terms), we choose our ordering as $y < \dot{y} < \ddot{y} < \dddot{y} < P < \dot{P} < H < \dot{H} < V < \dot{V}$. The system then becomes the following differential polynomials:

$$\dot{H} + \alpha P, \qquad \text{leader}\dot{H}, \qquad \text{(S21)}$$
$$\dot{V} - \lambda P + \eta V + \beta HV, \qquad \text{leader}\dot{V}, \qquad \text{(S22)}$$
$$H(\dot{P} + \alpha P) - \beta H^2 V + \alpha\gamma P^2, \qquad \text{leader}V, \qquad \text{(S23)}$$
$$y - V, \qquad \text{leader}V. \qquad \text{(S24)}$$

Reduce equation (S9) and equation (S10) by equation (S11) to get

$$\dot{H} + \alpha P, \qquad \text{leader}\dot{H}, \qquad \text{(S25)}$$
$$\dot{y} - \lambda P + \eta y + \beta Hy, \qquad \text{leader}H, \qquad \text{(S26)}$$
$$H(\dot{P} + \alpha P) - \beta H^2 y + \alpha\gamma P^2, \qquad \text{leader}H^2, \qquad \text{(S27)}$$
$$y - V, \qquad \text{leader}V. \qquad \text{(S28)}$$

The derivative of equation (S13) is

$$\ddot{y} - \lambda\dot{P} + \eta\dot{y} + \beta\dot{H}y + \beta H\dot{y}. \qquad \text{(S13')}$$

Reduce equation (S12) by equation (S13') then by equation (S13), and reduce equation (S14) by equation (S13) to get

$$-\ddot{y}y + \lambda y\dot{P} - \lambda\dot{y}P + \dot{y}^2 + \alpha\beta y^2 P, \qquad \text{leader}\dot{P}, \qquad \text{(S29)}$$

$$\dot{y} - \lambda P + \eta y + \beta H y \qquad \text{leader} H, \tag{S30}$$
$$(-\dot{y} + \lambda P - \eta y)(\dot{P} + \alpha P) - (-\dot{y} + \lambda P - \eta y)^2 + \alpha\beta\gamma y P^2, \qquad \text{leader} \dot{P}, \tag{S31}$$
$$y - V, \qquad \text{leader} V. \tag{S32}$$

Next, reduce equation (S18) by equation (S16), creating equation (S20) with leader P^2.

$$\begin{aligned} &P^2\Big(\lambda^2\dot{y} + \alpha\beta\gamma\lambda y^2 - \alpha\beta\lambda y^2 + \alpha\lambda^2 y + \lambda^3(-y)\Big) \\ &+ P\Big(\lambda y\ddot{y} + \alpha\beta y^2\dot{y} - \alpha\lambda y\dot{y} - \eta\lambda y\dot{y} + 2\lambda^2 y\dot{y} \\ &- 2\lambda\dot{y}^2 + \alpha\beta\eta y^3 - \alpha\eta\lambda y^2 + 2\eta\lambda^2 y^2\Big) \\ &- \eta y^2\ddot{y} - 2\eta\lambda y^2\dot{y} + \eta y\dot{y}^2 - \lambda y\dot{y}^2 \\ &+ \dot{y}^3 - y\dot{y}\ddot{y} - \eta^2\lambda y^3 \end{aligned} \tag{S20}$$

We take its derivative.

$$\begin{aligned} &\dot{P}\Big(2P\left(\lambda^2\dot{y} + \alpha\beta\gamma\lambda y^2 - \alpha\beta\lambda y^2 + \alpha\lambda^2 y + \lambda^3(-y)\right) + \lambda y\ddot{y} + \alpha\beta y^2\dot{y} \\ &- \alpha\lambda y\dot{y} - \eta\lambda y\dot{y} + 2\lambda^2 y\dot{y} - 2\lambda\dot{y}^2 + \alpha\beta\eta y^3 - \alpha\eta\lambda y^2 + 2\eta\lambda^2 y^2\Big) \\ &+ P^2\Big(\lambda^2\ddot{y} + 2\alpha\beta\gamma\lambda y\dot{y} - 2\alpha\beta\lambda y\dot{y} + \alpha\lambda^2\dot{y} + \lambda^3\left(-\dot{y}\right)\Big) \\ &+ P\Big(\lambda y\dddot{y} + \alpha\beta y^2\ddot{y} - \alpha\lambda y\ddot{y} - \eta\lambda y\ddot{y} + 2\lambda^2 y\ddot{y} + 3\alpha\beta\eta y^2\dot{y} \\ &+ 2\alpha\beta y\dot{y}^2 - 2\alpha\eta\lambda y\dot{y} - \alpha\lambda\dot{y}^2 + 4\eta\lambda^2 y\dot{y} - \eta\lambda\dot{y}^2 + 2\lambda^2\dot{y}^2 - 3\lambda\dot{y}\ddot{y}\Big) \\ &- \eta y^2\dddot{y} - 2\eta\lambda y^2\ddot{y} - y\ddot{y}^2 - 3\eta^2\lambda y^2\dot{y} - 4\eta\lambda y\dot{y}^2 + \eta\dot{y}^3 \\ &- \lambda\dot{y}^3 - y\dddot{y}\dot{y} - 2\lambda y\dot{y}\ddot{y} + 2\dot{y}^2\ddot{y} \end{aligned} \tag{S20'}$$

The terms of the pseudodivision become too long to continue writing, so we continue the remaining reductions in Mathematica only. Due to the length of the resulting input-output relation, the coefficients are listed, in descending order (as per our ordering):

Coefficient of $\dddot{y}^2$: $\gamma\lambda^2\left(\dot{y} + \eta y\right)\left(\lambda\dot{y} + \alpha\beta(\gamma-1)y^2 + \lambda(\alpha-\lambda)y\right)$

Coefficient of $\dddot{y}$: $\alpha^3\beta^3(\gamma-1)\eta^2 y^6$

$$\begin{aligned} &- \alpha^2\beta^2\eta^2\lambda(-4\lambda\gamma^2 + 2\alpha\gamma + 4\lambda\gamma - 3\alpha + \lambda)y^5 \\ &+ \alpha\beta\eta^2\lambda^2((\gamma-3)\alpha^2 + 2(2\gamma\lambda + \lambda)\alpha - 4\gamma\lambda^2)y^4 - \alpha^2\eta^2\lambda^3(\lambda-\alpha)y^3 \\ &+ 2\alpha\beta\gamma(2\gamma-3)\lambda^2\dot{y}^4 \\ &+ \Big(\left(-(\gamma-2)\eta\alpha + \gamma\lambda\alpha - \alpha^2(\gamma-1) + \eta(-2\gamma\eta + \eta + \gamma\lambda)\right)\lambda^3 \\ &- \alpha\beta\Big(\alpha(3\gamma+2) + (-6\gamma^2 + 6\gamma + 2)\eta + \gamma(2\gamma-9)\lambda\Big)y\lambda^2 \\ &- \alpha^2\beta^2(4\gamma^2 - 4\gamma - 1)y^2\lambda\Big)\dot{y}^3 + \Big[\alpha^3\beta^3(\gamma-1)y^4 \\ &- \alpha^2\beta^2\lambda\Big(-4\lambda\gamma^2 + 4\lambda\gamma + \alpha(2\gamma-3) + (4\gamma^2 - 2\gamma - 4)\eta + \lambda\Big)y^3 \\ &+ \alpha\beta\lambda^2\Big((\gamma-3)\alpha^2 + 2(-4\eta + 2\gamma\lambda + \lambda)\alpha + 3(\gamma-1)\eta^2 - 4\gamma\lambda^2 \end{aligned}$$

$$+ 2(-2\gamma^2 + 7\gamma + 1)\eta\lambda\Big) y^2 - \lambda^3\Big(- \alpha^3 + \big(2(\gamma-2)\eta + \lambda\big)\alpha^2$$
$$+ (\gamma-1)\eta(3\eta - 2\lambda)\alpha + (1-3\gamma)\eta^2\lambda\Big) y\Big]\dot{y}^2$$
$$+ \Big((1-3\gamma)\dot{y}\lambda^3 - \alpha\beta(2\gamma^2 - 3\gamma + 1)y^2\lambda^2$$
$$- \lambda^3\big(\alpha(2\gamma-1) + \gamma\eta - 2\gamma\lambda + \lambda\big)y\Big)\ddot{y}^2$$
$$+ \Big[2\alpha^3\beta^3(\gamma-1)\eta y^5 - \alpha^2\beta^2\eta\lambda\big(\alpha(4\gamma-6) + (2\gamma-3)\eta$$
$$+ 2(-4\gamma^2 + 4\gamma + 1)\lambda\big)y^4 + \alpha\beta\eta\lambda^2\big(2(\gamma-3)\alpha^2 + 3(\gamma-2)\eta\alpha$$
$$+ 4(2\gamma+1)\lambda\alpha - 8\gamma\lambda^2 + (-2\gamma^2 + 5\gamma + 2)\eta\lambda\big)y^3$$
$$- \alpha\eta\lambda^3\big(- 2\alpha^2 + (\gamma-3)\eta\alpha + 2\lambda\alpha - (\gamma-2)\eta\lambda\big)y^2\Big]\dot{y}$$
$$+ (2\alpha^2\beta^2(2\gamma^2 - 3\gamma + 1)\eta\lambda y^4$$
$$- \alpha\beta\eta\lambda^2(\alpha(4-6\gamma) + \gamma\eta + 4\gamma\lambda - 2\lambda)y^3$$
$$- \eta\lambda^3(-2\alpha^2 - \gamma\eta\alpha + 2\lambda\alpha + 2\gamma\eta\lambda)y^2$$
$$+ (\lambda^3(-2\alpha(\gamma-1) + (2-3\gamma)\eta + \gamma\lambda) - \alpha\beta(4\gamma^2 - 7\gamma + 2)\lambda^2 y)\dot{y}^2$$
$$+ ((2\alpha^2 + (4-5\gamma)\eta\alpha - 2\lambda\alpha - 2\eta\lambda + \gamma\eta(\eta + 3\lambda))y\lambda^3$$
$$- \alpha(\alpha\beta(4-6\gamma) + 2\beta(2\gamma-1)(2(\gamma-1)\eta + \lambda))y^2\lambda^2$$
$$+ 2\alpha^2\beta^2(2\gamma^2 - 3\gamma + 1)y^3\lambda)\dot{y})\ddot{y}$$

Coefficient of $\ddot{y}^4$: $(-1+2\gamma)\lambda^3$

Coefficient of $\ddot{y}^3$: $\lambda\Big[\lambda\dot{y}\Big(\lambda(3\alpha(\gamma-1) + 3\gamma\eta - 2\gamma\lambda - 2\eta + \lambda)$

$$+ \alpha\beta\left(6\gamma^2 - 12\gamma + 5\right)y\Big) - 2\alpha^2\beta^2\gamma\left(2\gamma^2 - 3\gamma + 1\right)y^3$$
$$- \lambda^2 y\left(\alpha^2(2\gamma-1) + \alpha(-(\gamma-2)\eta - 2\gamma\lambda + \lambda) + \eta(\gamma(\eta - 2\lambda) + \lambda)\right)$$
$$- \alpha\beta\lambda y^2\left(\alpha\left(6\gamma^2 - 5\gamma + 1\right) + \left(-4\gamma^2 + 5\gamma - 2\right)\eta + 2\gamma(1-2\gamma)\lambda\right)\Big]$$

Coefficient of $\ddot{y}^2$: $\dot{y}^2(\lambda^3(\alpha^2(3\gamma-4) + \alpha(\gamma-1)(4\eta - 3\lambda)$

$$+ \eta((2\gamma-1)\eta + (3-5\gamma)\lambda)) - \alpha^2\beta^2(-12\gamma^3 + 28\gamma^2 - 22\gamma + 7)\lambda y^2$$
$$- \alpha\beta\lambda^2 y(\alpha(-12\gamma^2 + 18\gamma - 11) + (\gamma^2 + 7\gamma - 8)\eta + 6\gamma^2\lambda))$$
$$+ \dot{y}(-\alpha^3\beta^3(\gamma-1)\gamma y^4 + \alpha^2\beta^2\lambda y^3(\alpha(6\gamma^2 - 9\gamma + 2)$$
$$+ (-10\gamma^2 + 17\gamma - 8)\eta + (-4\gamma^3 + 4\gamma^2 + 2\gamma - 1)\lambda)$$
$$- \alpha\beta\lambda^2 y^2(\alpha^2(\gamma^2 - 9\gamma + 4) + \alpha(6\gamma^2 + 5\gamma - 10)\eta$$
$$+ \alpha(4\gamma^2 + 6\gamma - 3)\lambda - 2(5\gamma^2 - 7\gamma + 1)\eta\lambda + (1 - 4\gamma^2)\lambda^2 - 2(\gamma+1)\eta^2)$$
$$- \lambda^3 y(\alpha^3(\gamma-2) + \alpha^2(2(\gamma+1)\eta - (\gamma-2)\lambda) + \alpha\eta((2-3\gamma)\lambda + 2\eta)$$
$$+ 2(1-2\gamma)\eta^2\lambda)) + \alpha\beta(-5\gamma^2 + 9\gamma - 2)\lambda^2\dot{y}^3$$
$$- \alpha^3\beta^3(\gamma-1)\gamma\eta y^5 + \alpha^2\beta^2\eta\lambda y^4(\alpha(6\gamma^2 - 9\gamma + 2)$$
$$+ (-4\gamma^3 + 4\gamma^2 + 2\gamma - 1)\lambda + (\gamma-1)\eta) - \alpha\beta\eta\lambda^2 y^3(\alpha^2(\gamma^2 - 9\gamma + 4)$$
$$+ \alpha((4\gamma^2 + 6\gamma - 3)\lambda + 2(\gamma-1)\eta) + \lambda((-4\gamma^2 + 8\gamma - 2)\eta - 4\gamma^2\lambda + \lambda))$$
$$- \eta\lambda^3 y^2(\alpha^3(\gamma-2) + \alpha^2(-\gamma\eta - \gamma\lambda + \eta + 2\lambda) + 2\alpha\eta\lambda + \gamma\eta^2\lambda)$$

Coefficient of $\ddot{y}$: $\alpha^3\beta^3(\gamma-1)\eta^2(\alpha+4\gamma\lambda-2\lambda)y^6+\alpha^2\beta^2\eta^2\lambda((3-2\gamma)\alpha^2$
$+(4\gamma^2+2\gamma-5)\lambda\alpha-\lambda(\eta+4\gamma\lambda-2\lambda))y^5+\alpha\beta\eta^2\lambda^2((\gamma-3)\alpha^3$
$+4(\gamma+1)\lambda\alpha^2-2\lambda(-\eta+2\gamma\lambda+\lambda)\alpha-4\gamma\eta\lambda^2)y^4$
$-\alpha^2\eta^2\lambda^3(-\alpha^2+\lambda\alpha+\eta\lambda)y^3+(-\alpha^2(12\gamma^3-34\gamma^2+27\gamma-4)\lambda y\beta^2$
$-\alpha\lambda^2(2\alpha(3\gamma^2-5\gamma+2)+(2\gamma^2-7\gamma+4)\eta+\gamma(5-4\gamma)\lambda)\beta)\dot{y}^4$
$+((((\gamma-3)\alpha^3+(4(\gamma-1)\eta-(\gamma-3)\lambda)\alpha^2+\eta((2\gamma-1)\eta$
$+(6-5\gamma)\lambda)\alpha+(3-5\gamma)\eta^2\lambda)\lambda^3+\alpha\beta((2\gamma^2-17\gamma+9)\alpha^2$
$+6(2\gamma^2-\gamma+1)\eta\alpha+4\gamma(\gamma+1)\lambda\alpha+(3-8\gamma)\eta^2$
$-4(\gamma-1)\gamma\lambda^2+3\gamma(1-4\gamma)\eta\lambda)y\lambda^2-\alpha^2\beta^2(3\alpha(4\gamma^2-7\gamma+3)$
$+(-16\gamma^2+18\gamma+2)\eta+(-8\gamma^3+24\gamma^2-21\gamma+3)\lambda)y^2\lambda$
$-\alpha^3\beta^3(-2\gamma^2+5\gamma-3)y^3)\dot{y}^3$
$+(\alpha^3\beta^3(\gamma-1)(\alpha+2\gamma\eta-6\eta+4\gamma\lambda-2\lambda)y^4-\alpha^2\beta^2\lambda((2\gamma-3)\alpha^2$
$+2(6\gamma^2-12\gamma+7)\eta\alpha+(-4\gamma^2-2\gamma+5)\lambda\alpha-3(\gamma-2)\eta^2$
$+2(2\gamma-1)\lambda^2+(-8\gamma^3+40\gamma^2-46\gamma+9)\eta\lambda)y^3$
$-\alpha\beta\lambda^2(-2((\gamma^2-8\gamma+5)\eta+2(\gamma+1)\lambda)\alpha^2+((\gamma-7)\eta^2+4(\gamma-2)\lambda\eta$
$+2(2\gamma+1)\lambda^2)\alpha-\alpha^3(\gamma-3)+\eta\lambda((4\gamma^2-3\gamma-2)\eta-4\gamma\lambda+2\lambda))y^2$
$-\lambda^3(-\alpha^4+(2\eta+\lambda)\alpha^3+\eta(2\gamma\eta+\eta-\lambda)\alpha^2$
$-(2\gamma+1)\eta^2\lambda\alpha+(1-3\gamma)\eta^3\lambda)y)\dot{y}^2+$
$(\alpha^3\beta^3(\gamma-1)\eta(2\alpha-3\eta+8\gamma\lambda-4\lambda)y^5+\alpha^2\beta^2\eta\lambda((6-4\gamma)\alpha^2$
$+((3\gamma-5)\eta+2(4\gamma^2+2\gamma-5)\lambda)\alpha$
$+\lambda((-16\gamma^2+25\gamma-7)\eta-8\gamma\lambda+4\lambda))y^4+\alpha\beta\eta\lambda^2(2(\gamma-3)\alpha^3$
$+(\gamma+1)(\eta+8\lambda)\alpha^2-2\lambda((2\gamma^2+4\gamma-5)\eta+2(2\gamma+1)\lambda)\alpha$
$+\eta\lambda((\gamma+2)\eta+2(2\gamma^2-2\gamma-1)\lambda))y^3$
$+\alpha\eta\lambda^3(2\alpha^3+(-\gamma\eta+\eta-2\lambda)\alpha^2+(\gamma-3)\eta\lambda\alpha+(\gamma-2)\eta^2\lambda)y^2)\dot{y}$

Coefficient of $\dot{y}^6$: $\alpha^2\beta^2(3-2\gamma)^2\gamma\lambda$

Coefficient of $\dot{y}^5$: $\alpha\beta\Big[-\lambda^2\Big(\alpha^2\Big(\gamma^2-7\gamma+2\Big)+\alpha\left(6\gamma^2-5\gamma+4\right)\eta+$
$5\alpha\gamma\lambda-\gamma^2\lambda^2+(2-4\gamma)\eta^2+\gamma(5-6\gamma)\eta\lambda\Big)$
$-\alpha^2\beta^2\left(\gamma^2-3\gamma+2\right)y^2-\alpha\beta\lambda y\Big(-2\alpha\left(3\gamma^2-5\gamma+2\right)$
$+\Big(6\gamma^2-5\gamma-4\Big)\eta+4\gamma(\gamma-2)^2\lambda\Big)\Big]$

Coefficient of $\dot{y}^4$: $\lambda^3\left(-\alpha^2+\alpha(\gamma-2)\eta-(1-2\gamma)\eta^2\right)\left(\alpha^2-\alpha\lambda-\eta\lambda\right)$
$-\alpha^3\beta^3y^3\left(\alpha(\gamma-1)+\left(\gamma^2-5\gamma+4\right)\eta+\left(4\gamma^2-3\gamma-2\right)\lambda\right)$
$-\alpha^2\beta^2\lambda y^2\Big[\alpha^2(3-2\gamma)+\alpha\left(-6\gamma^2+11\gamma-6\right)\eta+\alpha\left(4\gamma^2-2\gamma+3\right)\lambda$
$+\left(4\gamma^3-28\gamma^2+31\gamma+3\right)\eta\lambda+2(\gamma-2)\eta^2+4(\gamma-3)\gamma\lambda^2\Big]$
$-\alpha\beta\lambda^2y\Big[\alpha^3(\gamma-3)+\alpha^2\gamma((\gamma-5)\eta+5\lambda)$

$$+\alpha\Big(\gamma(7-4\gamma)\eta\lambda-4\gamma\lambda^2+3\eta^2\Big)+\gamma\eta\lambda((\gamma-4)\lambda-2(\gamma-3)\eta)\Big]$$

Coefficient of $\dot{y}^3$: $\alpha y\Big[-\eta\lambda^3((\gamma-2)\eta-2\alpha)\left(-\alpha^2+\alpha\lambda+\eta\lambda\right)$

$$+\alpha^3\beta^4(\gamma-1)y^4-\alpha^2\beta^3y^3\Big(2\alpha(\gamma-1)\eta+\alpha(2\gamma-3)\lambda$$
$$+\left(8\gamma^2-3\gamma-8\right)\eta\lambda+\left(-4\gamma^2+4\gamma+1\right)\lambda^2-2(\gamma-1)\eta^2\Big)$$
$$-\alpha\beta^2\lambda y^2\Big(\alpha^2((6-4\gamma)\eta-(\gamma-3)\lambda)+\alpha\Big(2\left(4\gamma^2-4\gamma+7\right)\eta\lambda$$
$$+(\gamma-2)\eta^2-2(2\gamma+1)\lambda^2\Big)+\lambda\Big(\left(-12\gamma^2+12\gamma+7\right)\eta^2$$
$$+2\left(6\gamma^2-16\gamma-1\right)\eta\lambda+4\gamma\lambda^2\Big)\Big)$$
$$-\beta\lambda^2y\Big(\alpha^3(2(\gamma-3)\eta-\lambda)+\alpha^2\left(2(\gamma-1)\eta^2+(11\gamma-4)\eta\lambda+\lambda^2\right)$$
$$+\alpha\eta\lambda\left(\left(-4\gamma^2+5\gamma-3\right)\eta-8\gamma\lambda+2\lambda\right)$$
$$+\eta^2\lambda\left(\gamma^2\lambda+3\gamma\eta-8\gamma\lambda+\lambda\right)\Big)\Big]$$

Coefficient of $\dot{y}^2$: $\alpha\eta y^2\Big[\alpha\eta\lambda^3\left(-\alpha^2+\alpha\lambda+\eta\lambda\right)+3\alpha^3\beta^4(\gamma-1)y^4$

$$-\alpha^2\beta^3y^3\Big(\alpha(\gamma-1)\eta+3\alpha(2\gamma-3)\lambda+\left(4\gamma^2+3\gamma-10\right)\eta\lambda$$
$$+3\left(-4\gamma^2+4\gamma+1\right)\lambda^2\Big)+\alpha\beta^2\lambda y^2\Big(\alpha^2((2\gamma-3)\eta+3(\gamma-3)\lambda)$$
$$+\alpha\lambda\left(\left(-4\gamma^2+10\gamma-19\right)\eta+6(2\gamma\lambda+\lambda)\right)$$
$$+\lambda\left(4\left(-3\gamma^2+7\gamma+1\right)\eta\lambda+(3\gamma-4)\eta^2-12\gamma\lambda^2\right)\Big)$$
$$-\beta\lambda^2y\Big(\alpha^3((\gamma-3)\eta-3\lambda)+\alpha^2\lambda((7\gamma-8)\eta+3\lambda)$$
$$+\alpha(\gamma-1)\eta\lambda(3\eta-4\lambda)-\left(\gamma^2+4\gamma-1\right)\eta^2\lambda^2\Big)\Big]$$

Coefficient of $\dot{y}$: $\alpha^2\beta\eta^2y^4\Big[-\lambda^3\left(-3\alpha^2+\alpha(\gamma-4)\eta+3\alpha\lambda+2\eta\lambda\right)$

$$+3\alpha^2\beta^3(\gamma-1)y^3+\beta\lambda^2y\Big(3\alpha^2(\gamma-3)+4\alpha(\gamma-2)\eta+6\alpha(2\gamma+1)\lambda$$
$$+2\lambda\left(-2\gamma^2\eta+4\gamma\eta-6\gamma\lambda+\eta\right)\Big)$$
$$+\alpha\beta^2\lambda y^2\left(\alpha(9-6\gamma)+3\left(4\gamma^2-4\gamma-1\right)\lambda+(4-3\gamma)\eta\right)\Big]$$

Coefficient of y^8: $\alpha^4\beta^4(\gamma-1)\eta^3$

Coefficient of y^7: $\alpha^3\beta^3\eta^3\lambda\left(\alpha(3-2\gamma)+\left(4\gamma^2-4\gamma-1\right)\lambda\right)$

Coefficient of y^6: $\alpha^2\beta^2\eta^3\lambda^2\left(\alpha^2(\gamma-3)+2\alpha(2\gamma\lambda+\lambda)-4\gamma\lambda^2\right)$

Coefficient of y^5: $-\alpha^3\beta\eta^3\lambda^3(\lambda-\alpha)$

There are far too many coefficients of our input-output relation to consider all at once,

so we pick a choice few to consider.

$$\text{Coefficient of } \ddot{y}^4\text{: } \gamma\lambda^2\left(\dot{y}+\eta y\right)\big(\lambda\dot{y}+\alpha\beta(\gamma-1)y^2+\lambda(\alpha-\lambda)y\big)$$
$$\text{Coefficient of } y^5\text{: } -\alpha^3\beta\eta^3\lambda^3(\lambda-\alpha)$$

We construct an exhaustive summary of this portion and have the coefficients

$$\eta, \qquad \lambda, \qquad \alpha\beta(\gamma-1), \qquad \lambda(\alpha-\lambda), \qquad \alpha^3\beta\eta^3\lambda^3(\lambda-\alpha).$$

We evaluate the above coefficients at a symbolic parameter value $p = [a, b, g, e, l]$ (corresponding to $[\alpha, \beta, \gamma, \eta, \lambda]$), giving us the following equations.

$$\eta - e = 0, \qquad \lambda - l = 0, \qquad \alpha\beta(\gamma-1) - ab(g-1) = 0, \qquad \lambda(\alpha-\lambda) - l(a-l) = 0,$$
$$\alpha^3\beta\eta^3\lambda^3(\lambda-\alpha) - a^3be^3l^3(l-a) = 0.$$

Obviously $\eta = e$ and $\lambda = l$ (uniquely). We can find α, β, and γ by solving (in order)

$$\lambda(\alpha-\lambda) - l(a-l) = 0,$$
$$\alpha^3\beta\eta^3\lambda^3(\lambda-\alpha) - a^3be^3l^3(l-a) = 0, \text{ and}$$
$$\alpha\beta(\gamma-1) - ab(g-1) = 0,$$

to uniquely obtain $\alpha = a$, $\beta = b$, and $\gamma = g$.

Thus, since our parameters have a single unique solution, our model is globally identifiable.

Supplementary Figure S6: Dynamics of the macroparasite model incorporating the innate immune response

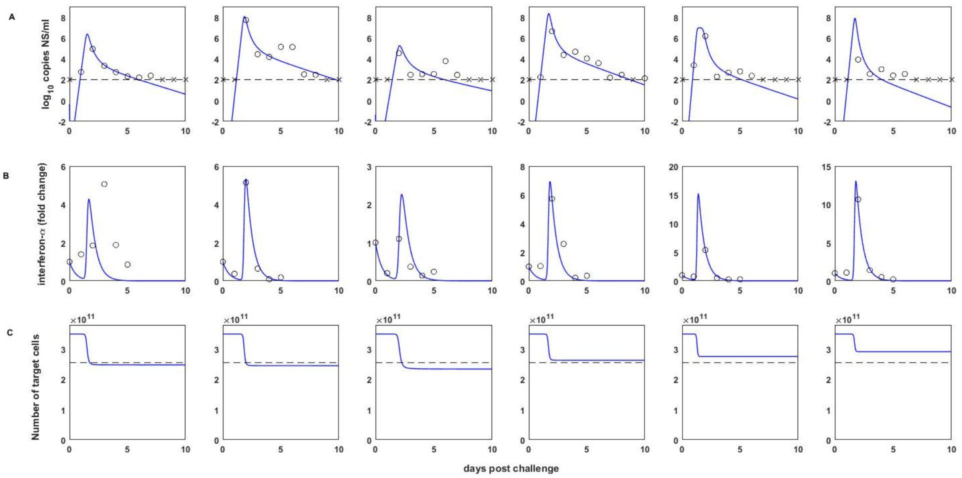

Figure 6. Dynamics of only the parameterized macroparasite model incorporating the innate immune response. Dynamics are as in Figure 4 of the main text.

Supplementary References

1. Roberts MG, Smith G, Grenfell BT. (1995) Mathematical models for macroparasites of wildlife. Ecology of Infectious Diseases in Natural Populations. pp. 177–208.

2. Dou D, Hernández-Neuta I, Wang H, Östbye H, Qian X, Thiele S, et al. (2017) Analysis of IAV Replication and Co-infection Dynamics by a Versatile RNA Viral Genome Labeling Method. Cell Reports. 20: 251–263. doi:10.1016/j.celrep.2017.06.021

3. Clapham HE, Tricou V, Van Vinh Chau N, Simmons CP, Ferguson NM. (2014) Within-host viral dynamics of dengue serotype 1 infection. Journal of The Royal Society Interface. 11: 20140094–20140094. doi:10.1098/rsif.2014.0094

4. Ben-Shachar R, Schmidler S, Koelle K. Drivers of Inter-individual Variation in Dengue Viral Load Dynamics. (2016) PLOS Computational Biology. 12: e1005194. doi:10.1371/journal.pcbi.1005194

5. Saenz RA, Quinlivan M, Elton D, MacRae S, Blunden AS, Mumford JA, et al. (2010) Dynamics of influenza virus infection and pathology. J Virol. 84: 3974–3983. doi:10.1128/JVI.02078-09
